# circNOX4 activates an inflammatory fibroblast niche to promote tumor growth and metastasis in NSCLC via FAP/IL-6 axis

**DOI:** 10.1186/s12943-024-01957-5

**Published:** 2024-03-08

**Authors:** Yan Zhao, Yunlong Jia, Jiali Wang, Xiaolin Chen, Jingya Han, Shuman Zhen, Shuxian Yin, Wei Lv, Fan Yu, Jiaqi Wang, Fan Xu, Xinming Zhao, Lihua Liu

**Affiliations:** 1 Department of Tumor Immunotherapy, The Fourth Hospital of Hebei Medical University and Hebei Provincial Tumor Hospital, Shijiazhuang, 050035 China; 2 Department of Oncology, The Fourth Hospital of Hebei Medical University and Hebei Provincial Tumor Hospital, Shijiazhuang, 050011 China; 3 Department of Nuclear Medicine, The Fourth Hospital of Hebei Medical University and Hebei Provincial Tumor Hospital, Shijiazhuang, 050011 China; 4 Department of Thoracic Surgery, The Fourth Hospital of Hebei Medical University and Hebei Provincial Tumor Hospital, Shijiazhuang, 050011 China; 5 Departments of Oncology, The Affiliated Hospital of Chengde Medical University, Chengde, 067000 China; 6 Hebei Provincial Key Laboratory of Tumor Microenvironment and Drug Resistance, Shijiazhuang, 050011 China; 7 Cancer Research Institute of Hebei Province, Shijiazhuang, 050011 China; 8 International Cooperation Laboratory of Stem Cell Research, Hebei Medical University, Shijiazhuang, 050011 China

**Keywords:** NSCLC, CAFs, circNOX4, FAP, IL-6, Metastasis

## Abstract

**Background:**

Cancer-associated fibroblasts (CAFs) orchestrate a supportive niche that fuels cancer metastatic development in non-small cell lung cancer (NSCLC). Due to the heterogeneity and plasticity of CAFs, manipulating the activated phenotype of fibroblasts is a promising strategy for cancer therapy. However, the underlying mechanisms of fibroblast activation and phenotype switching that drive metastasis remain elusive.

**Methods:**

The clinical implications of fibroblast activation protein (FAP)-positive CAFs (FAP^+^CAFs) were evaluated based on tumor specimens from NSCLC patients and bioinformatic analysis of online databases. CAF-specific circular RNAs (circRNAs) were screened by circRNA microarrays of primary human CAFs and matched normal fibroblasts (NFs). Survival analyses were performed to assess the prognostic value of circNOX4 in NSCLC clinical samples. The biological effects of circNOX4 were investigated by gain- and loss-of-function experiments in vitro and in vivo. Fluorescence in situ hybridization, luciferase reporter assays, RNA immunoprecipitation, and miRNA rescue experiments were conducted to elucidate the underlying mechanisms of fibroblast activation. Cytokine antibody array, transwell coculture system, and enzyme-linked immunosorbent assay (ELISA) were performed to investigate the downstream effectors that promote cancer metastasis.

**Results:**

FAP^+^CAFs were significantly enriched in metastatic cancer samples, and their higher abundance was correlated with the worse overall survival in NSCLC patients. A novel CAF-specific circRNA, circNOX4 (hsa_circ_0023988), evoked the phenotypic transition from NFs into CAFs and promoted the migration and invasion of NSCLC in vitro and in vivo. Clinically, circNOX4 correlated with the poor prognosis of advanced NSCLC patients. Mechanistically, circNOX4 upregulated FAP by sponging miR-329-5p, which led to fibroblast activation. Furthermore, the circNOX4/miR-329-5p/FAP axis activated an inflammatory fibroblast niche by preferentially inducing interleukin-6 (IL-6) and eventually promoting NSCLC progression. Disruption of the intercellular circNOX4/IL-6 axis significantly suppressed tumor growth and metastatic colonization in vivo.

**Conclusions:**

Our study reveals a role of the circRNA-induced fibroblast niche in tumor metastasis and highlights that targeting the circNOX4/FAP/IL-6 axis is a promising strategy for the intervention of NSCLC metastasis.

**Supplementary Information:**

The online version contains supplementary material available at 10.1186/s12943-024-01957-5.

## Background

Metastasis is the leading cause of cancer-related mortality and consists of multistep processes driven by the cooperation of cancer cells with the tumor microenvironment (TME) [1]. As predominant stromal components in the TME, cancer-associated fibroblasts (CAFs), also known as activated fibroblasts, actively orchestrate a supportive niche as “fertilized soil” and endow incipient cancer cells with traits needed to develop metastases [2]. CAFs are also identified as a generalized biomarker of the fibrotic TME subtype that correlates with inferior therapeutic outcomes and prognosis across various cancers [3]. With the development of single-cell sequencing approaches, tremendous insight has been yielded into the heterogeneity and plasticity of CAFs. The composition and functional states of fibroblasts differ extensively in the evolving TME, which makes targeting CAFs challenging in clinical settings [4–6]. Therefore, reprogramming CAFs into a “normal” or quiescent state might be a promising approach that benefits early cancer treatment and inhibits metastasis.

Accumulating evidence has implied that aberrant fibroblast activation is an early event before cancer cell dissemination [7]. When the interaction with cancer cells begins, local normal fibroblasts (NFs) are usually among the first cell types to be recruited and activated into CAFs, and ultimately establish a niche to facilitate metastatic cascades [8, 9]. Previous studies have shown that paracrine signaling molecules drive fibroblasts from homeostasis to activated states, including transforming growth factor β (TGF-β), platelet-derived growth factor (PDGF), fibroblast growth factor (FGF), and interleukin-1 [7]. Moreover, direct physical contact with cancer cells via ligand receptor binding also leads to the transition of NFs into CAFs [10]. More interestingly, CAFs maintain activated phenotypes even in the absence of cancer cells [11]. However, the underlying mechanisms of fibroblast activation and phenotype switching that drive metastasis have not been extensively elucidated.

Fibroblast activation protein (FAP), a type II membrane serine protease, is widely acknowledged as one of the canonical CAF markers [12]. FAP is overexpressed on activated fibroblasts and coincides with poor prognosis in cancers, which has made FAP-targeted imaging and therapy appealing [13]. Indeed, over 28 different cancer entities have been accurately diagnosed in patients utilizing FAP ligands, including metastatic lesions [14]. Cumulative studies have revealed that FAP-positive CAFs (FAP^+^CAFs) account for the main phenotypes of activated fibroblasts in the TME, and they execute crucial functions in promoting tumor growth, angiogenesis and metastasis, as well as the formation and maintenance of an immunosuppressive microenvironment [15–17]. In addition, our previous work demonstrated that FAP orchestrated the immune microenvironment and served as a biomarker of immunotherapy resistance. Patients with high FAP expression had a significantly shorter progression-free survival in immunotherapy of non-small cell lung cancer (NSCLC) [18]. However, the upstream epigenetic mechanisms by which FAP drives tumor progression are unclear and warrant further investigation.

In this study, we determined that FAP^+^CAFs are correlated with metastasis and poor prognosis in NSCLC patients. By investigating the critical mediator in fibroblast activation, we identified a novel circular RNA (circRNA), circNOX4, that is significantly upregulated in CAFs and correlates with the worse overall survival of NSCLC patients. Importantly, circNOX4 fuels tumor growth and metastasis by activating the fibroblast niche via the miR-329-5p/FAP/IL-6 axis. Our results elucidate a novel mechanism of fibroblast activation and IL-6 signaling by CAFs to establish an inflammatory niche and highlight circNOX4 as a promising candidate for CAF-targeted therapy in NSCLC.

## Materials and methods

### Patients and clinical samples

Tumor samples were collected from a cohort of 84 NSCLC patients at the Fourth Hospital of Hebei Medical University (Shijiazhuang, China) between August 2018 and March 2019 with the approval of the Medical Ethics Committee and informed consent from the corresponding participants (2018MEC005). All samples were histologically confirmed to have NSCLC. Patients’ clinical information was collected and stored in a database, which was updated every 3 months by telephone follow-up. Overall survival (OS) was defined as the time interval from diagnosis to the date of cancer-related death or the last follow-up (January 2024).

### Immunohistochemistry (IHC)

IHC staining was performed according to standard protocols as previously described [18]. The staining was evaluated based on the intensity of FAP (ab207178, 1:250, Abcam, CA, USA) by two independent pathologists, and all scoring was completed blinded to the clinical information. The intensity of FAP was classified as 0, no staining; 1, weak intensity; 2, moderate intensity; and 3, high intensity. Scores of 0 and 1 were considered low expression, while scores of 2 and 3 were considered high expression.

### Isolation, identification, and culture of fibroblasts from human NSCLC tissues

Five paired CAFs and NFs were isolated from human NSCLC tissues and normal tissues (more than 5 cm away from the tumor margin). Freshly isolated surgical specimens were acquired from patients with the approval of The Fourth Hospital of Hebei Medical University (2018MEC005). The patient’s basic information is listed in Additional file: Table S1. For fibroblast isolation, tissue was initially stored and washed in PBS with penicillin/streptomycin (100 U/100 μg, BI, Israel). The tissue was then physically minced and digested enzymatically in a solution containing 2 mg/mL DNase (Gibco, Waltham, MA, USA), 5 mg/mL collagenase type I (Gibco, Waltham, MA, USA), 100 U/mL penicillin, and 100 μg/mL streptomycin at 37 °C for 2 h. After a series of filtration and centrifugation steps, cells were subsequently cultured in 15% fetal bovine serum (FBS, Gibco, Waltham, MA, USA), DMEM (Gibco, Waltham, MA, USA), 20 ng/mL EGF (PeproTech, Suzhou, China) and penicillin/streptomycin (100 U/100 μg) at 37 °C in humidified air with 5% CO_2_ for subsequent expansion. Fibroblast populations were identified by morphology, western blotting and immunofluorescence staining (fibroblast markers α-SMA and FAP; epithelial cell marker EpCAM). Fibroblasts were subcultured at 80% confluence and used in experiments at passages 4–10. Antibody information is provided in Additional file: Table S2.

### Cell lines

Human lung cancer cell lines (A549, PC9, H226, and H1581) and human bronchial epithelial cells (BEAS-2B) were purchased from Procell Life Science & Technology Co., Ltd. (Wuhan, China) and maintained in RPMI-1640 medium (Gibco, Waltham, MA, USA) containing 10% FBS, 100 U/ml penicillin, and 100 μg/ml streptomycin at 37 °C and 5% CO_2_ under saturated humidity.

### circRNA microarray

The circRNA microarray analysis (GEO accession number: GSE244065) was performed by the Sinotech Genomics Corporation, Shanghai, China. In brief, total RNA from three paired sets of CAFs and NFs was extracted by TRIzol reagent (Life Technologies, Carlsbad, CA, US) and purified using the RNeasy Mini Kit (Qiagen, GmBH, Germany). Then, RNA samples were utilized to generate biotinylated cRNA targets for the Sino Human ceRNA array V3.0. After hybridization, slides were screened by the Agilent Microarray Scanner (Agilent Technologies, Santa Clara, CA, US). Raw data were extracted and normalized, and circRNAs with a fold change of at least 1.5 and *P* values < 0.05 were selected for further analysis by R software.

### Confirmation of the circular structure

Both complementary DNA (cDNA) and genomic DNA (gDNA) extracted from CAFs and NFs were amplified with circNOX4 primers (both convergent and divergent primers). Agarose gel electrophoresis was applied to analyze the RT–PCR products. The back-splice junction site of circNOX4 was verified by Sanger sequencing. Extracted RNAs were treated with RNase R (Geneseed, 3 U/μg, 37 °C, 20 min) to detect the stability of circRNA.

### RNA isolation, cDNA synthesis, and quantitative RT-PCR (qRT-PCR) assay

In brief, total RNA was isolated using the E.Z.N.A. Total RNA Kit (OMEGA, Japan). cDNA was synthesized using HiScript III RT SuperMix (Vazyme, Nanjing, China) and amplified using a Light Cycler 480 II Real-Time PCR System (Roche, Basel, Switzerland) with ChamQ Universal SYBR qPCR Master Mix (Vazyme, Nanjing, China). GAPDH and U6 were used as internal controls via the classical ΔΔCt method. Primers were designed and synthesized by RiboBio (Guangzhou, China) and Tsingke (Wuhan, China). The primer sequences are listed in Additional file: Table S3.

### RNA-fluorescence in situ hybridization (FISH)

FISH assays were performed on CAFs/NFs and NSCLC tissues as previously described [19]. FISH probes were designed and synthesized by Servicebio (Wuhan, China). Dig-labeled probes specific for circNOX4 and biotinylated locked nucleic acid miR-329-5p probes were used during hybridization. The targeted sequences of the probes are provided in Additional file: Table S4. The FISH score for circNOX4 in NSCLC tissues was determined based on the integrated optical density values scanned by AIPATHWELL software (Servicebio Technology Co., Wuhan, China). The optimal cut-off value of 0.1 was determined by X-tile software. Based on this threshold value, patients were categorized into circNOX4 high-expression or low-expression subgroups for further analysis.

### Collagen contraction assay

The fibroblasts were embedded in three-dimensional collagen matrices in 24-well plates with Rat Tail Collagen I (Corning, NY, USA) at a final concentration of 2 mg/mL collagen and 5 × 10^5^ cells per mL. A 500 μL aliquot of this mixture was dispensed into each well and allowed to solidify for 30 min at 37 °C. Gels were freed from the wells using a pipette tip after polymerization, and complete culture medium was added to the wells, followed by incubation for 12 h. ImageJ software was employed to measure the contractile capacity, which is defined by the following equation: [Area (well)-Area (gel)/Area (well)] × 100%.

### Western blotting analysis

Standard western blotting was carried out by a wet transfer system (Bio-Rad Laboratories). Briefly, cells were lysed in lysis buffer (Solarbio, Beijing, China) supplemented with protease inhibitors. Total proteins were separated on SDS–PAGE gels and then transferred onto PVDF membranes (Millipore, Billerica, MA, USA). After blocking the membranes with 5% milk-TBST, they were incubated with appropriate dilutions of specific primary antibodies against FAP (ab207178, 1:1000, Abcam) and α-SMA (ab124964, 1:1000, Abcam). The blots were incubated with HRP-conjugated secondary antibodies and visualized using the ECL system (GE, Boston, MA, USA).

### Cell transfection

Indicated small interfering RNAs (siRNAs) were transfected into primary fibroblasts or cancer cells using Lipofectamine 3000 reagent (Invitrogen, Carlsbad, CA, USA). For stable transfection, the lentiviral vector (pGLV3/GFP/Puro) containing short hairpin RNAs (shRNAs) was designed based on the si-circNOX4#1 sequence and packaged by GenePharma (Shanghai, China). The circNOX4 sequence was packaged into the pGLV5/GFP/Puro vector. Fluorescent signals were observed using a fluorescence microscope (Olympus, Japan). The miRNA mimics, inhibitors and corresponding negative controls (NC) for miR-329-5p and miR-624-5p were synthesized and purchased from RiboBio (Guangzhou, China). The sequences used are listed in Additional file: Table S5.

### Cell viability assay

The cell viability of fibroblasts was measured by a cell counting kit-8 (CCK-8) assay (Solarbio, Beijing, China). A total of 2000 cells were seeded in 96-well plates per well, and 10 μl of CCK-8 reagent was added and incubated for 2 h at 37 °C. The absorbance was detected using a microplate reader (Tecan, Switzerland) at a wavelength of 450 nm.

### Coculture of CAFs and cancer cells, conditioned medium (CM) preparation, migration and invasion assays

For coculture of CAFs and cancer cells, NSCLC cells were plated in the upper chamber of transwell apparatus (0.4 μm insert; Corning, Corning, NY, USA), and CAFs were cultured in the lower chamber. After incubation for 48 h, supernatants were collected for further measurements. For CM preparation, indicated fibroblasts were cultured to reach 80% confluence and then replaced with serum-free DMEM for 48 h. The supernatants were collected and centrifuged to remove cell pellets. The CM was subjected to cytological experiments or stored at -80 °C.

For the cell migration assay, NSCLC cells were cultured in six-well plates and scraped linearly to create an artificial wound. Then, NSCLC cells were incubated in CAF-CM or NF-CM for 48 h. For the cell invasion assay, the upper chambers of the Transwell plates (8 μm insert) were precoated with Matrigel (dilution, 1:8; BD Biosciences, Franklin Lakes, NJ, USA) at 37 °C for 30 min. NSCLC cells (5 × 10^4^/well) suspended in serum-free medium were seeded into the upper chambers. Indicated fibroblasts (3 × 10^4^/well) were seeded into the lower chambers. Subsequent procedures were performed according to previous studies [20].

### Dual luciferase reporter assay

Wild-type (WT) or mutant (MUT) pmiRGlo-circNOX4 and pmiRGlo-FAP-3′UTR dual luciferase reporter vectors incorporating predicted binding sites for miR-329-5p and miR-624-5p were constructed by GenePharma (Shanghai, China). The sequences are provided in Additional file: Table S5 and Additional file: Table S6. Cells were seeded on 12-well plates at a density of 60% and then co-transfected with WT or MUT plasmids with miRNA mimics or a negative control (NC) using Lipofectamine 3000 reagent. After 48 h of incubation, the cells were collected and tested for luciferase activity with a Dual-Luciferase Assay System kit (Promega, Madison, WI, USA) according to the manufacturer’s instructions.

### RNA immunoprecipitation (RIP) assay

RIP was performed with an RNA immunoprecipitation kit (Geneseed, Guangzhou, China). CAFs were transfected with miR-NC or miR-329-5p for 48 h. Then, the cells were lysed with RIP lysis buffer and incubated with magnetic beads precoated with antibodies against Ago2 (ab186733, 1:50, Abcam) or IgG (ab172730, 1:50, Abcam). After the antibody was recovered by protein A/G beads and purification was completed, qRT-PCR was performed to detect the enrichment of circNOX4 and miR-329-5p in the precipitates.

### Cytokine antibody array

A human cytokine array (AAH-CYT-G5; RayBiotech, Norcross, GA, USA) was used to measure the secretion levels of 80 cytokines in the CM of sh-circNOX4 CAFs and sh-NC CAFs according to the manufacturer’s instructions. Quantitative array analysis was performed using the ImageQuant LAS4000 Scanner (GE, Boston, MA, USA). Cytokines were screened using the following integrated conditions: Fold change ≥ 1.2 and fluorescence intensity values > 300.

### Enzyme-linked immunosorbent assay (ELISA)

Cytokines in the supernatant of fibroblasts or cancer cells were measured using ELISA kits (IL-6, CCL2: Abcolonal, Wuhan, China; TGF-β2, IGFBP-4: RayBiotech, Norcross, GA, USA) following the manufacturer’s instructions.

### Mouse experiments

The animal experiments were conducted in accordance with national guidelines and approved by the Animal Research Committee of the Fourth Hospital of Hebei Medical University (SYXK2022-011). BALB/c nude mice (female, age 5 weeks) were purchased from Beijing HFK Bio-Technology Co., Ltd.

For the subcutaneous xenograft model, 5 × 10^6^ A549 cells alone or in a 3:1 mixture with stably transfected fibroblasts were subcutaneously implanted into the axillae of mice (*n* = 6–8 mice per group). For the anti-IL-6 experiment group, neutralizing anti-IL-6 antibody (10 μg/ml, R&D, Minneapolis, MN, USA) was administered via intratumoral injection twice a week after 14 days of model construction [21]. Tumor volume was calculated by using the formula: Volume = (length × width^2^)/2. IHC analysis was performed on the xenografts to evaluate protein expression utilizing antibodies against FAP (ab207178, 1:250, Abcam), CD31 (28083–1-AP, 1:200, Proteintech), N-cadherin (Proteintech, 1:100, Servicebio), Vimentin (10366–1-AP, 1:400, Proteintech), matrix metalloproteinase 2 (MMP2) (ab86607, 1:200, Abcam), matrix metalloproteinase 9 (MMP9) (ab76003, 1:200, Abcam) and IL-6 (GB11117, 1:200, Servicebio).

For the lung metastasis model, 2 × 10^5^ A549 cells in 100 μL PBS were injected into the tail vein of BALB/c nude mice. The mice were randomly assigned to one of three groups: (1) untreated A549 cells, (2) A549 cells "primed" in vitro for 72 h in a transwell coculture model with CAFs transduced with empty vector (sh-NC), or (3) CAFs transduced with circNOX4 shRNA (sh-circNOX4). After 5 weeks, the mice were sacrificed and dissected, and the lungs were collected. Hematoxylin and eosin (HE) staining was used to observe the metastatic foci.

### ^68^ Ga-labeled FAPI-04 PET imaging of mice

The preparation of ^68^ Ga-Labeled FAPI-04 solutions was performed by previous method [22]. Briefly, ^68^ Ga was generated by a ^68^Ge–^68^ Ga generator and eluted with a solution of 0.1 M hydrochloride. The mixture of the ^68^ Ga solution (74 MBq), 1.0 M sodium acetate (95 μL) and 1.15 mM FAPI-04 (20 μL) were reacted at 95 °C for 10 min. ^68^ Ga FAPI-04 PET imaging was performed up to 60 min after intravenous injection of 11.1 MBq of ^68^ Ga-FAPI-04 in tumor xenograft mice (*n* = 3) using a micro-PET/SPECT/CT machine (Novel Medical Equipment Ltd., China) under isoflurane anesthesia. PET images were reconstructed with the comprehensive image analysis software Pmod v4.201 (PMOD Technologies LLC, Switzerland) and were converted to SUV images. Quantification was performed using a region-of-interest (ROI) technique and expressed as SUVmax.

### Statistical analyses

Statistical analyses were performed using SPSS 22.0 software (SPSS Inc., Chicago, IL, USA), GraphPad Prism 7 (GraphPad, San Diego, CA, USA) and R software V4.2.0 (RStudio, Murray Hill, NJ, USA). The data are presented as the mean ± SD of at least three biological replicates. Differences between the two groups were analyzed by t-tests. The associations between circNOX4 expression and clinicopathological characteristics were evaluated by the Chi-square test. The statistical significance of survival was estimated by the Kaplan–Meier method and Cox analysis. *P* values less than 0.05 were considered statistically significant.

## Results

### FAP^+^CAFs correlate with tumor metastasis and poor survival in NSCLC patients

**Fig. 1:**
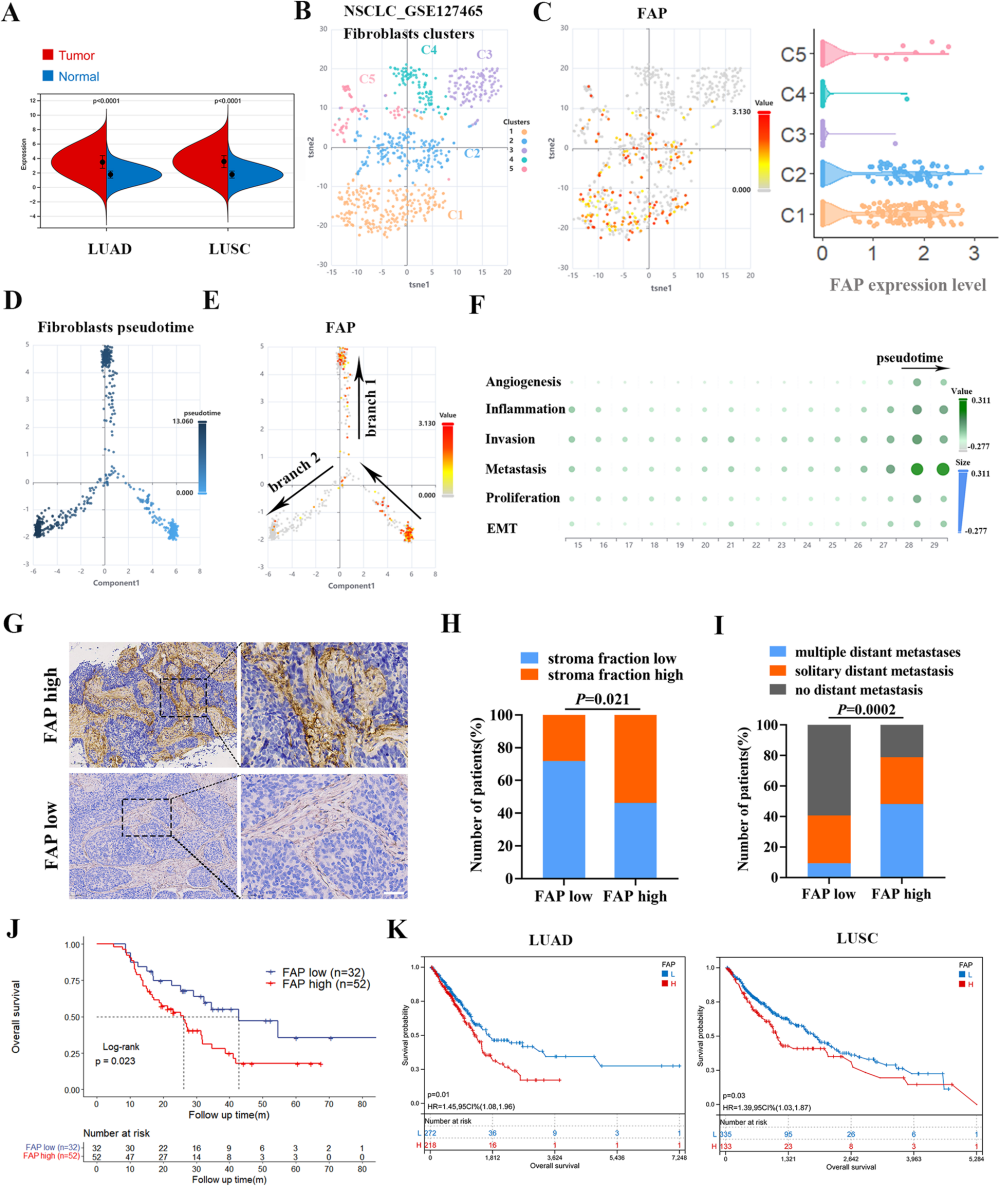
FAP expression in the TME of NSCLC and its clinical significance. **A** The mRNA expression levels of FAP in tumor tissues and normal tissues in the LUAD and LUSC cohorts from the TCGA database. **B** Distinct fibroblast clusters in NSCLC _GSE127465 database as visualized by single-cell analysis. **C** The expression of FAP in different fibroblast clusters. **D** The cell development pseudotime constructed by CellTracer tools (http://bio-bigdata.hrbmu.edu.cn/CellTracer). The cells were ordered in a pseudo-temporal manner using Monocle2. **E** FAP expression in cellular developmental pseudotime in different branches. **F** The dynamic variation in functional activities along developmental pseudotime of branch 1. **G** Representative images of immunohistochemistry for high- or low-FAP expression in NSCLC tissues. (brown = FAP; blue = hematoxylin nuclear staining). Scale bar = 50 μm. **H** The proportion of patients with high/low stroma fraction in high/low FAP expression groups. **I** The proportion of patients with different metastasis status in high/low FAP expression groups. **J** Kaplan–Meier curves with patients stratified according to FAP expression (low vs. high) for OS. **K** Kaplan–Meier curves for LUAD and LUSC patients according to FAP expression levels in the TCGA database

Given the supporting role of CAFs in tumor growth and metastasis [7], we first confirmed the contribution of FAP^+^CAFs in NSCLC. We queried FAP mRNA expression using the TCGA database and found that FAP was upregulated in lung adenocarcinoma (LUAD) and lung squamous cell carcinoma (LUSC) compared with normal lung tissues (Fig. 1A). The data also showed the copy number and mutation status of FAP gene were not correlated with FAP mRNA expression, indicating that the high expression level of FAP was potentially regulated by epigenetic mechanisms (Fig. S1A). Next, we analyzed the spatial cellular expression pattern of FAP across different datasets and cell types in the TME and confirmed that FAP was specifically enriched in fibroblast lineages (Fig. S1B). Moreover, we analyzed the GEO profile GSE127465, which revealed that fibroblasts were distributed into five clusters in NSCLC. We found that FAP was enriched in the C1 and C2 subpopulations, indicating the heterogeneous nature of CAFs in the TME (Fig. 1B, C). To uncover the biological function of FAP^+^CAFs in development trajectories, we constructed developmental pseudotime and discovered that FAP expression was relatively higher along the branch 1 cellular state, which was accompanied by increased protumor activities such as angiogenesis, inflammation, and metastasis (Fig. 1D-F). These findings revealed that FAP exhibits cell lineage- and developmental stage-dependent expression patterns while undergoing protumor activities.

To investigate the clinical relevance between fibroblast activation and the prognosis of NSCLC patients, we collected clinical samples from 84 NSCLC patients and performed IHC staining. The result showed that FAP was expressed in the stromal compartment in NSCLC tissues (Fig. 1G). Analysis of clinicopathological variables based on FAP expression level revealed that high expression of FAP was positively correlated with high stromal fraction and multiple distant metastases (Fig. 1H, I), indicating that FAP is closely related to the process of metastasis. Moreover, FAP expression was inversely correlated with overall survival in NSCLC patients (*P* = 0.023; Fig. 1J), in line with the clinical outcome data from the TCGA database (Fig. 1K). Taken together, FAP^+^CAFs are populated in metastatic tumors with inferior clinical outcomes in NSCLC patients.

### Identification of circNOX4, a CAF-specific circRNA, that predicts poor prognosis for NSCLC patients

**Fig. 2:**
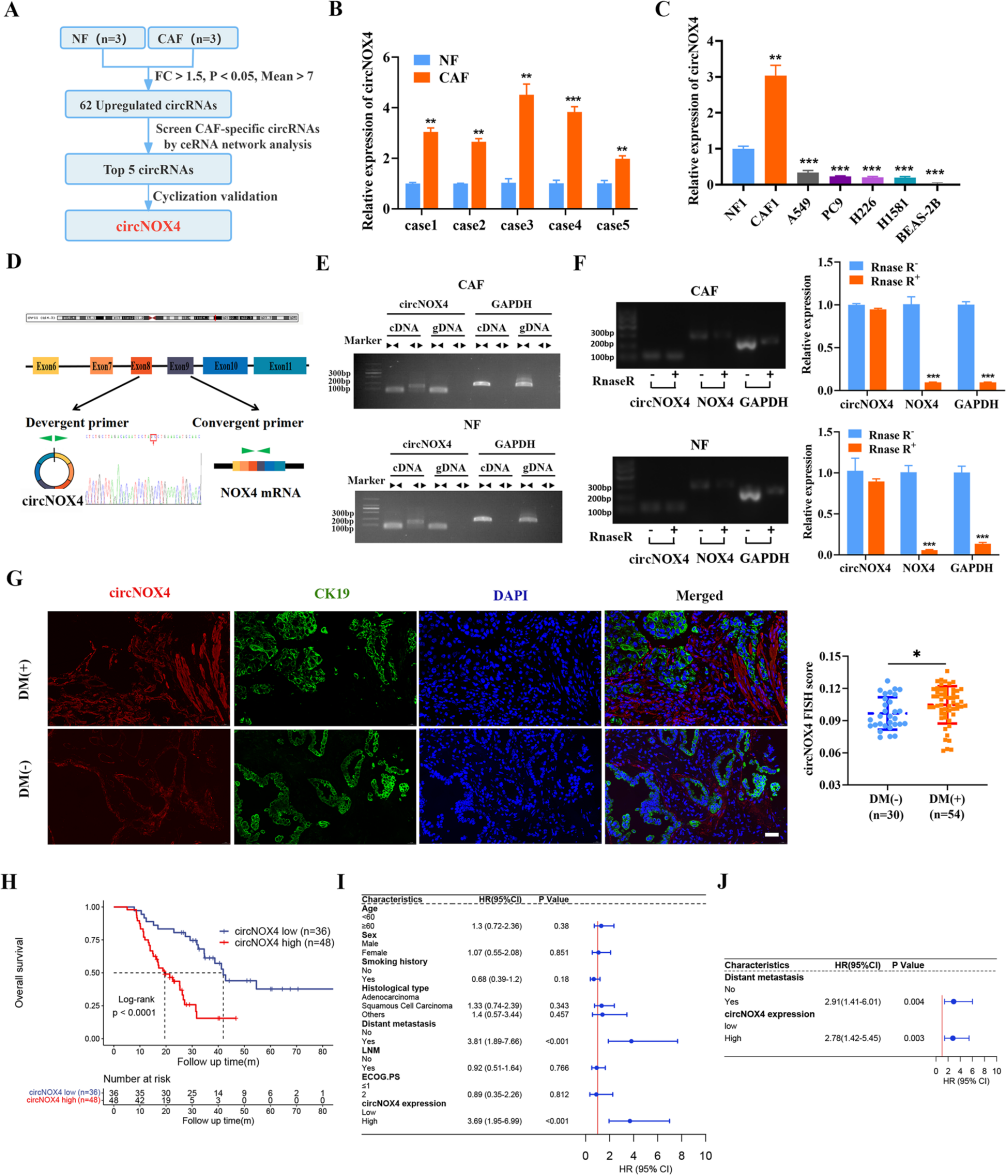
circNOX4 is upregulated in CAFs and correlates with poor prognosis of NSCLC patients.** A** Schematic illustration of the identification of circNOX4 upregulated in CAFs. **B** qRT-PCR analysis of circNOX4 expression in five paired CAFs and NFs. **C** qRT-PCR analysis of circNOX4 expression in CAFs, NFs, NSCLC cell lines (A549, PC9, H226, H1581), and human bronchial epithelial cells (BEAS-2B). **D** Diagram illustrating the genomic location and back-splicing mode of circNOX4 from the NOX4 host gene. The head-to-tail splice junction site was confirmed by Sanger sequencing using the divergent primer. **E** cDNA and gDNA of CAFs and NFs were amplified with convergent and divergent primers. GAPDH was the negative control. **F** PCR and qRT-PCR analysis of circNOX4, linear NOX4, and GAPDH in CAFs and NFs with or without RNase R treatment. **G** Representative images (left) and quantification (right) of circNOX4 by using RNA-FISH in NSCLC tissues with and without distant metastasis. CK19 indicated cancer cells. The nucleus (blue) was stained with DAPI. Scale bar = 40 μm, DM, distant metastasis.** H** Kaplan–Meier curves of OS for NSCLC patients with high (*n* = 48) and low (*n* = 36) circNOX4 expression. The cut-off value was calculated using X-tile. Statistical significance was determined by the log-rank test. **I** Forest plots of OS, as determined by the univariate Cox proportional hazards model. **J** Forest plots of OS, as determined by the multivariate Cox proportional hazards model, were constructed using variables significantly associated with OS by the univariate Cox proportional hazards model (*P* < 0.1). Data are expressed as mean ± SD and statistical significance was determined by two-tailed Student’s t-tests and log-rank tests. *P < 0.05, **P < 0.01 and ***P < 0.001

To gain insight into the contribution of circRNAs to aberrant fibroblast activation in the TME, we first isolated primary CAFs and NFs from human NSCLC tissues and adjacent normal tissues and identified the characteristics of fibroblasts (Fig. S2A-C). By performing circRNA microarray screening of CAFs and NFs, we identified 62 upregulated circRNAs in CAFs (cutoff value: |Fold change|> 1.5, *P* values < 0.05, Mean > 7, Fig. 2A and Fig. S3A). Then, we selected the top 5 upregulated circRNAs as candidate circRNAs based on a competing endogenous RNA (ceRNA) network and detected 2 circRNAs (circ_0064142 and circ_0023988) that were enriched in CAFs through gel electrophoresis (Fig. S3A-C). However, Sanger sequencing of the reverse transcription product failed to confirm the circular splicing of circ_0064142, so we chose circ_0023988 (circNOX4) for further identification. Then, qRT-PCR analysis showed that circNOX4 was significantly upregulated in CAFs compared to NFs, human NSCLC cell lines (A549, PC9, H226 and H1581) and bronchial epithelial cells (BEAS-2B), indicating a potential role in fibroblast activation (Fig. 2B, C). Next, we identified the origin of circNOX4, which was generated from exons 6–11 of the NOX4 transcript (chr11:89,155,069–89185063) with a length of 476 nt. The back-splice junction site of circNOX4 was confirmed by Sanger sequencing (Fig. 2D). To further verify the circular property of circNOX4, the back-splice junction site of circNOX4 was amplified by RT–PCR using convergent and divergent primers, which only amplified a specific divergent band in cDNA but not in gDNA (Fig. 2E). Additionally, circNOX4 was RNase R-resistant, whereas the control linear NOX4 and GAPDH mRNAs were not (Fig. 2F). These results indicate that circNOX4 is a bona fide circRNA highly expressed in CAFs.

To determine whether circNOX4 was clinically relevant in NSCLC patients, we performed fluorescence in situ hybridization (FISH) in 84 NSCLC specimens. The results showed that circNOX4 was expressed in the intratumoral stroma but not in cancer cells (CK19-positive areas) (Fig. 2G). Additionally, a higher circNOX4 expression level was correlated with distant metastasis (Fig. 2G, Table 1). Next, we investigated the correlation between circNOX4 expression and patient survival. The overall survival of NSCLC patients with high circNOX4 expression was significantly shorter than that of patients with low circNOX4 expression (*P* < 0.0001; Fig. 2H). Univariate and multivariate Cox regression models revealed that high circNOX4 expression and distant metastasis were independent prognostic factors for NSCLC (Fig. 2I, J). Collectively, our findings suggest that a CAF-specific circRNA, circNOX4, correlates with metastatic progression and may be a prognostic marker in NSCLC patients.

**Table 1:** Clinicopathological parameters of NSCLC patients and correlation with circNOX4 expression (*n* = 84)

Clinicopathological parameter	N of cases	circNOX4 expression		*P* value
		Low	High	
Age at diagnosis				
< 60	32	11	21	0.218
≥ 60	52	25	27	
Sex				
Male	66	31	35	0.145
Female	18	5	13	
Smoking history				
Yes	55	24	31	0.842
No	29	12	17	
Histological type				
Adenocacinoma	42	17	25	0.806
Squamous cell carcinoma	34	16	18	
Others	8	3	5	
Distant metastasis				
Yes	54	16	38	0.001**
No	30	20	10	
LNM				
Yes	59	25	34	0.890
No	25	11	14	
ECOG PS				
≤ 1	76	32	44	0.720
2	8	4	4	

*LNM* Lymph node metastasis, *ECOG PS* Eastern Cooperative Oncology Group Performance Status

### circNOX4 contributes to tumor progression by mediating fibroblast activation

**Fig. 3:**
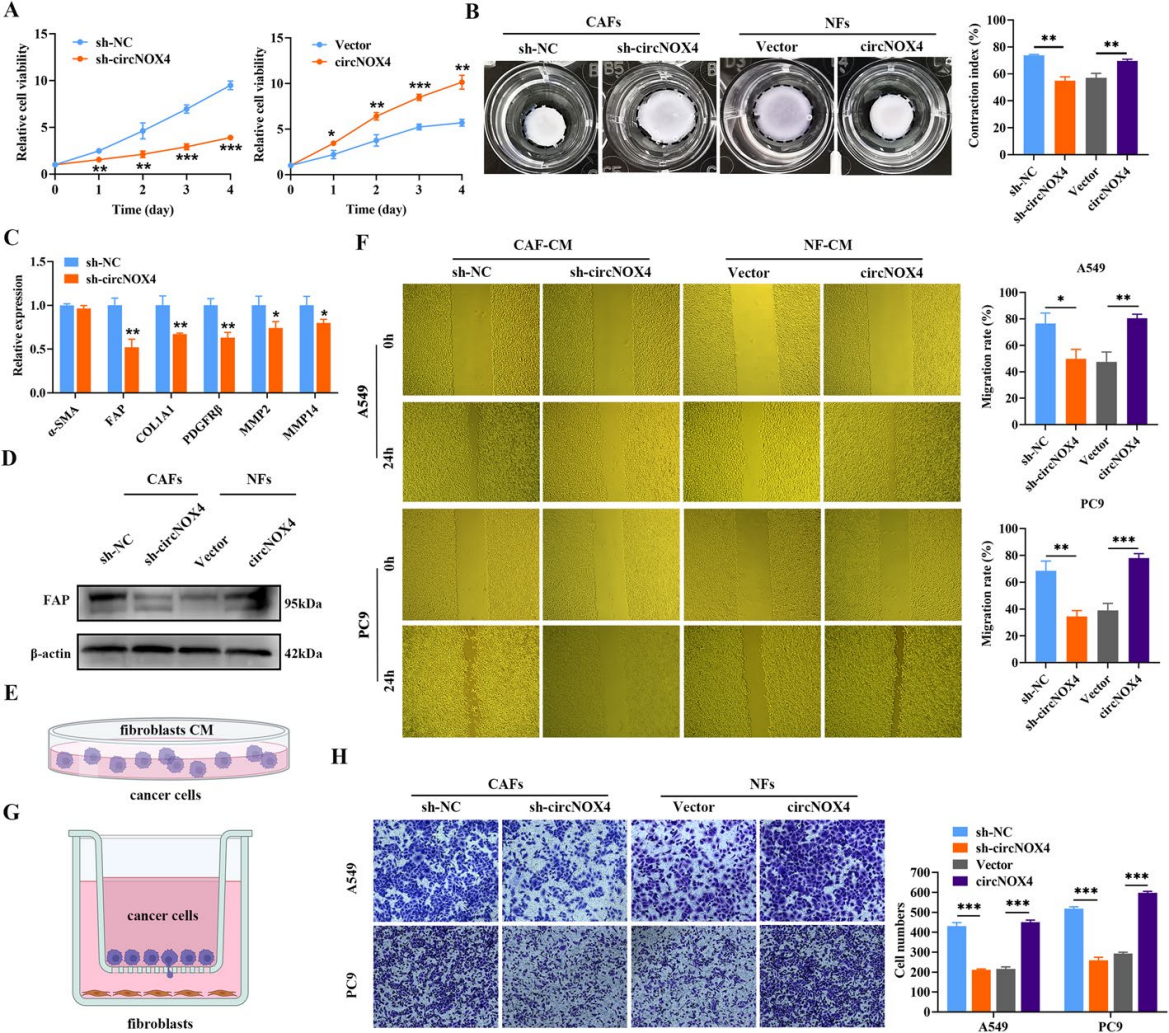
circNOX4 induces fibroblast activation and promotes the migration and invasion of NSCLC in vitro.** A** The proliferation of circNOX4-knockdown CAFs and circNOX4-overexpressing NFs as detected by CCK-8 assays. **B** Representative photographs of collagen gel contraction by the indicated CAFs and NFs. **C** The expression of CAF-related genes in sh-NC and sh-circNOX4 CAFs as detected by qRT-PCR.** D** Effects of circNOX4 on the expression of FAP by western blotting analysis. **E** Schematic representation showing that fibroblastic conditioned medium was added to NSCLC cells in wound healing assays. **F** Wound healing assays in A549 and PC9 cells treated with conditioned medium from circNOX4-knockdown CAFs or circNOX4-overexpressing NFs. **G** Schematic representation showing the establishment of the transwell coculture system. **H** Transwell coculture assays of invasion of A549 and PC9 cells with circNOX4-knockdown CAFs or circNOX4-overexpressing NFs. Data are expressed as mean ± SD from three independent experiments. Statistical analyses used the Student’s t-test. *P < 0.05, **P < 0.01, ***P < 0.001

To delineate the functional role of circNOX4 in the intrinsic characteristics of fibroblasts, we knocked down circNOX4 in CAFs or overexpressed circNOX4 in NFs without altering the expression of the parental gene NOX4 (Fig. S4A-C). Then, we speculated that circNOX4 could regulate the phenotype of fibroblasts. The CCK-8 and collagen contraction assays exhibited that circNOX4 overexpression significantly enhanced the proliferation and contraction capabilities of fibroblasts, while circNOX4 knockdown exerted the opposite effects (Fig. 3A, B). Afterwards, we examined how circNOX4 affected the expression of CAF markers and regulators, including FAP, MMP2, MMP14, collagen type 1 alpha 1 (COL1A1) and PDGFRβ. Interestingly, qRT-PCR suggested a reversion of the above gene expression profiles. Notable among them was FAP, which displayed the greatest reduction following circNOX4 knockdown (Fig. 3C). In parallel, circNOX4 overexpression upregulated FAP expression while circNOX4 knockdown attenuated FAP expression, as shown by western blotting and qRT-PCR analysis (Fig. 3D and Fig. S4D). To extend our understanding of circNOX4 in CAF activation, we detected circNOX4 expression in NFs after stimuli of TGF-β1, a critical mediator of fibroblast activation and cancer-stroma bidirectional communication within the TME (Fig. S5A). Consequently, the expression of circNOX4 and FAP increased in a dose- and time-dependent manner upon exposure to TGF-β1 (Fig. S5B, C). Collectively, these findings support a potent effect of circNOX4 on fibroblast activation.

As the activation of fibroblasts is linked to protumor properties, we explored the role of circNOX4 in supporting cancer cell migration and invasion. NSCLC cell lines were treated with fibroblast-derived conditioned medium (CAF-CM and NF-CM). As expected, the wound healing assays showed that medium from sh-circNOX4 CAFs drastically decreased the migration ability of A549 and PC9 cells. In stark contrast, overexpressing circNOX4 increased the migration ability of cancer cells (Fig. 3E, F). Meanwhile, the transwell coculture system demonstrated that sh-circNOX4 CAFs reduced the invasion of A549 and PC9 cells, while circNOX4-overexpressing NFs exerted the opposite effects (Fig. 3G, H). Overall, circNOX4 facilitates NSCLC migration and invasion by activating CAFs and maintaining a protumor phonotype.

### circNOX4 functions as a miR-329-5p sponge to upregulate FAP in CAFs

**Fig. 4:**
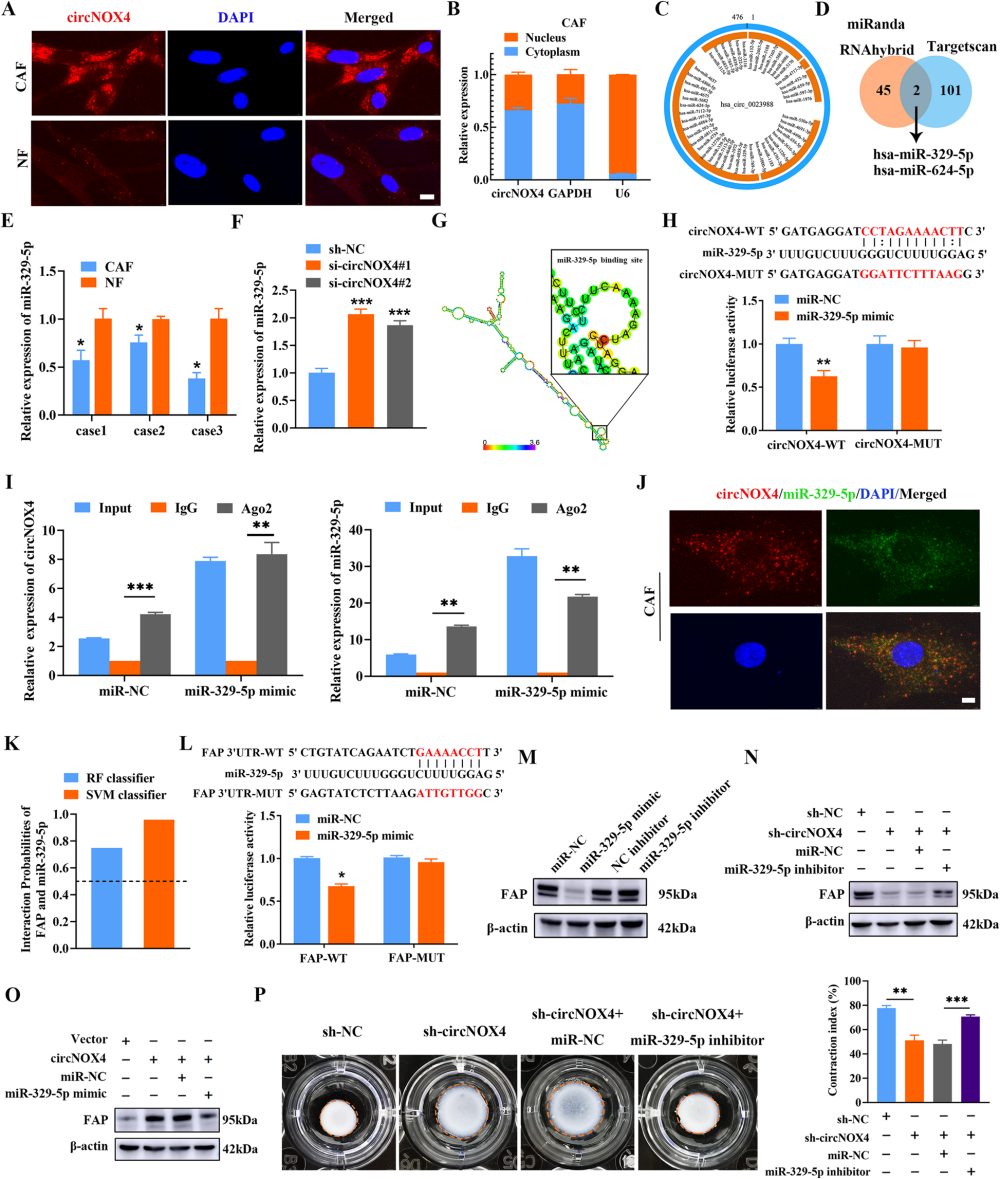
circNOX4 upregulates FAP expression by sponging miR-329-5p in CAFs.** A** FISH was performed to evaluate the sublocation of circNOX4 (red) in CAFs and NFs, respectively. The nucleus (blue) was stained with DAPI. Scale bar = 10 μm. **B** Expression of circNOX4 in either the nucleus or cytoplasm of CAFs. U6 and GAPDH were used as nuclear and cytoplasmic localization markers, respectively. **C** Schematic illustration of the potential target miRNAs of circNOX4 predicted by miRanda and RNAhybrid. **D** Venn analysis of the potential binding miRNAs of circNOX4 and FAP, predicted by miRanda, RNAhybrid, and TargetScan. **E** The expression of miR-329-5p in CAFs and NFs was analyzed by RT-qPCR. **F** The effect of circNOX4 siRNAs on the expression of miR-329-5p in CAFs. **G** Diagram of the secondary structure of circNOX4 and the possible binding sites with miR-329-5p predicted by RNAalifold. **H** The sequence alignment of circNOX4-WT and circNOX4-MUT in luciferase reporter assay for validating the binding of circNOX4 and miR-329-5p. Luciferase reporter assay showing the luciferase activity of the circNOX4 luciferase reporter plasmid (WT or MUT) following transfection with miR-NC or miR-329-5p mimic into CAFs.** I** Ago2-RIP assay was applied to detect the expression of circNOX4 and miR-329-5p in CAFs. **J** RNA-FISH was performed to evaluate the location of circNOX4 (red) and miR-329-5p (green) in CAFs. The nucleus (blue) was stained with DAPI. Scale bar = 10 μm. **K** The potential possibility of FAP binding to miR-329-5p was predicted by RNA–Protein Interaction Prediction (RPISeq) website. The interaction probabilities score ranged from 0 to 1 and > 0.5 were considered a close interaction. **L** The sequences of FAP 3'-UTR-WT and FAP 3'-UTR-MUT used in luciferase reporter assay were presented. Luciferase reporter assay showing the luciferase activity of the FAP 3'-UTR luciferase reporter plasmid (WT or MUT) following transfection with miR-NC or miR-329-5p mimic into CAFs. **M** Western blotting was used to investigate the effect of miR-329-5p on FAP expression. **N and O** The effect of the circNOX4/miR-329-5p axis on the expression level of FAP. **P** The effect of the circNOX4/miR-329-5p axis on the fibroblast activation reflected by collagen gel contraction. *P < 0.05, **P < 0.01, ***P < 0.001

To dissect the molecular mechanisms by which circNOX4 induces fibroblast activation, we detected its intracellular distribution. The results of FISH and subcellular fractionation assays showed that circNOX4 was mainly localized in the cytoplasm of CAFs and NFs (Fig. 4A, B). It has been reported that cytoplasmic circRNAs function by sponging miRNAs or encoding peptides [23]. We next observed that circNOX4 had a limited protein-coding potential with no predicted protein features using circRNADb software (R Score < 1.6, Fig. S6A). Therefore, we propose that miRNA sponge activity could be a possible mechanism for its functional effect.

To determine the miRNAs interacting with circNOX4, we overlapped the results obtained from two different algorithms (miRanda and RNAhybrid) predicting miRNAs that could bind with circNOX4 and screened 47 candidate miRNAs (Fig. 4C). Then, through prediction algorithms of the TargetScan database, only miR-329-5p and miR-624-5p were predicted to target both circNOX4 and FAP (Fig. 4D). We therefore explored the ceRNA network including circNOX4, miR-329-5p/miR-624-5p and FAP. The qRT-PCR results showed that miR-329-5p and miR-624-5p were downregulated in CAFs compared with paired NFs (Fig. 4E and Fig. S6B). Moreover, the expression of miR-329-5p and miR-624-5p was increased by knocking down circNOX4 (Fig. 4F and Fig. S6C), suggesting that circNOX4 could negatively regulate the expression of miR-329-5p and miR-624-5p.

Next, to validate whether circNOX4 sponges miR-329-5p and miR-624-5p, we constructed luciferase reporters harboring wild-type and mutant forms of circNOX4 according to the predicted binding sites on miR-329-5p or miR-624-5p, respectively (Fig. 4G, H and Fig. S6D). Consequently, the miR-329-5p mimic could specifically target the wild-type form of circNOX4, resulting in markedly decreased luciferase activity, but not the mutant form (Fig. 4H and Fig. S6E). RIP assays further confirmed the interaction of miR-329-5p with circNOX4 in CAFs (Fig. 4I). Moreover, RNA-FISH revealed that circNOX4 and miR-329-5p co-localized in the cytoplasm of CAFs (Fig. 4J). Unfortunately, due to the low abundance of circNOX4, AGO2 was unable to accumulate circNOX4 in NFs (Fig. S6F). In addition, the miR-624-5p mimic had no effect on the luciferase activity of the circNOX4 wild-type reporter (Fig. S6G). These results support that miR-329-5p directly binds to circNOX4 in CAFs, whereas miR-624-5p does not.

Furthermore, similar experiments were conducted to confirm the binding of miR-329-5p with the 3′-untranslated region (3′UTR) of FAP. As shown in Fig. 4K and Fig. 4L, we observed a high interaction probability between the miR-329-5p RNA sequence and FAP protein by the RPISeq prediction algorithm (https://www.pridb.gdcb.iastate.edu/RPISeq/), and the 3′UTR of FAP harbored specific sequences complementary to miR-329-5p. Dual-luciferase reporter assays showed decreased luciferase activity of the wild-type reporter of the FAP 3′UTR with the miR-329-5p mimic, while no significant differences were found with the mutant reporter (Fig. 4L). Additionally, the expression of FAP was significantly decreased with miR-329-5p mimic transfected into CAFs (Fig. 4M). The above evidence confirms that miR-329-5p binds to the 3' UTR of FAP in CAFs.

To verify whether circNOX4 regulated the expression of FAP through miR-329-5p, we performed rescue assays. Knockdown of circNOX4 significantly decreased the expression of FAP, while miR-329-5p inhibitor co-transfection neutralized this downregulation (Fig. 4N). Conversely, overexpression of circNOX4 significantly increased the expression of FAP, while miR-329-5p mimic co-transfection abrogated this alteration (Fig. 4O), indicating that the regulation of FAP by circNOX4 was dependent on miR-329-5p. In addition, we conducted collagen contraction assays to determine whether miR-329-5p was involved in circNOX4-induced fibroblast activation. The results showed that the inhibitory effect of circNOX4 knockdown was reversed by the miR-329-5p inhibitor (Fig. 4P). In summary, our results suggest that circNOX4 sponges miR-329-5p to upregulate FAP and induce fibroblast activation.

### circNOX4 facilitates CAF-mediated tumorigenesis and metastasis of NSCLC in vivo

**Fig. 5:**
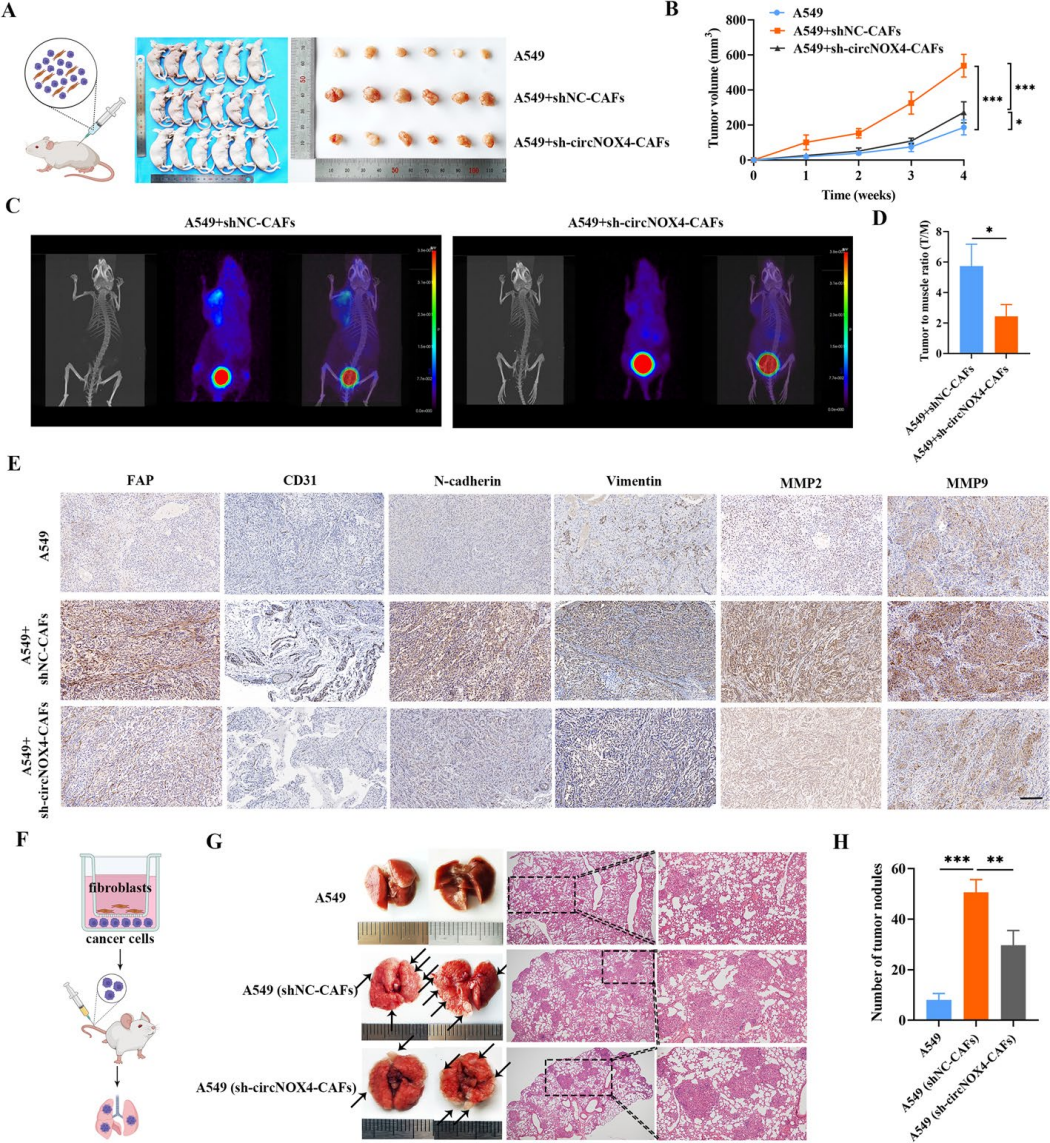
Knockdown of circNOX4 mitigates CAF-mediated tumor growth and metastasis of NSCLC in vivo. **A** Schematic illustration showing A549 cells were subcutaneously co-injected with or without CAFs stably transfected with sh-NC or sh-circNOX4 into nude mice (*n* = 6 per group). Images of nude mice and xenograft tumors from each group were shown in the right panel. **B** The volume of subcutaneous xenograft tumors (*n* = 6). **C** Representative images of micro-PET/CT imaging of the tumor-bearing nude mice after application of ^68^ Ga-FAPI-04. 3D MIP Images were collected over 1 h after the molecular imaging probe administration. **D** Quantified results from Micro-PET/CT images of the tumor to muscle ratio in each group. **E** Representative images of IHC for FAP, CD31, N-cadherin, Vimentin, MMP2, and MMP9 in xenograft tumors. Scale bar = 100 μm. **F** Schematic illustration of lung metastatic tumor model construction in nude mice. **G** The potential for lung metastasis was determined via vein tail injection of the indicated A549 cells. HE staining was used to observe the morphological changes in lung tissues. **H** Histogram analysis of the metastatic nodule number per lung. *P < 0.05, **P < 0.01, ***P < 0.001

To evaluate how circNOX4 contributes to the TME and cancer progression in vivo, we constructed two different mouse models: the xenograft tumor model and the lung metastasis model. For xenograft tumor model establishment, A549 cells were subcutaneously co-transplanted with or without CAFs isolated from NSCLC tissue samples (Fig. 5A). We found that xenografts derived from co-transplantation with CAFs resulted in enhanced tumor volume and tumor weight compared with xenografts derived from A549 cells alone, suggesting that CAFs promoted NSCLC progression (Fig. 5B, and Fig. S7A). More strikingly, sh-circNOX4 CAFs significantly stunted tumorigenesis compared with A549 cells co-transplanted with shNC-CAFs (Fig. 5B, and Fig. S7A), indicating that knocking down circNOX4 efficiently disabled CAFs and impeded the development of invasive tumors.

Next, to intuitively visualize the expression of FAP in vivo, we utilized micro-positron emission tomography (PET)/CT imaging with a specific nuclide molecular probe (^68^ Ga-FAPI-04). Micro-PET/CT revealed tracer uptake in the tumor xenografts, as shown in the 3D MIP images (Fig. 5C). The quantitative results of FAPI-PET imaging suggested that SUV uptake in the xenografts was significantly reduced in the sh-circNOX4 group compared to the control group (Fig. 5D), indicating that knocking down circNOX4 could downregulate FAP expression and reverse fibroblast activation in vivo. Furthermore, we examined the effect of circNOX4 on the expression of FAP, the endothelial marker CD31, mesenchymal markers (N-cadherin and Vimentin), and metastasis-related genes (MMP2 and MMP9). All these factors are critically involved in angiogenesis and epithelial-mesenchymal transformation (EMT), which contribute to the process of metastasis [24, 25]. The IHC results showed that CAFs significantly increased the expression levels of FAP, CD31, N-cadherin, Vimentin, and MMPs. However, these effects were reversed by transfecting sh-circNOX4 into CAFs, implying that circNOX4 provides a pro-tumorigenic niche that facilitates metastasis in vivo (Fig. 5E and Fig. S7B, C).

To further substantiate the effect of circNOX4, we constructed lung metastasis mouse models. In brief, A549 cells were either injected through the tail vein or "primed" in a coculture system with CAFs for 48 h in vitro prior to injection (Fig. 5F). As shown in Fig. 5G, more lung metastatic foci were detected in mice that had been injected with A549 cells "primed" with fibroblasts, compared with mice injected with the control A549 cells. Notably, a lower number of lung metastatic foci was presented in the sh-circNOX4-CAF group than in the shNC-CAF group, as evidenced by reduced HE-stained tumors in the lungs (Fig. 5G, H). All these data thus suggest that circNOX4 potentiates tumor metastasis by inducing fibroblast activation in vivo*.*

### The circNOX4/FAP axis establishes an inflammatory fibroblast niche by secreting IL-6

**Fig. 6:**
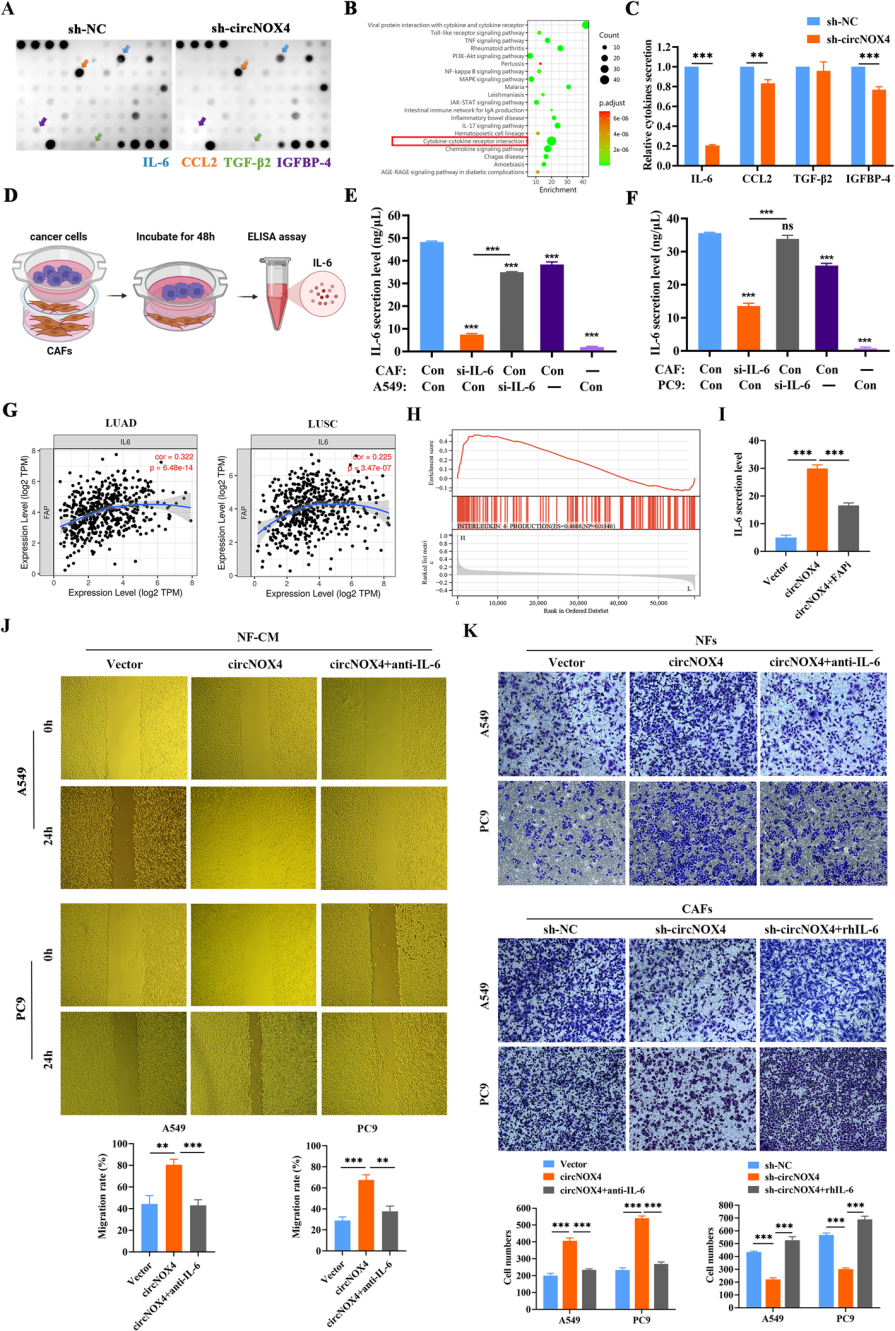
The circNOX4/FAP axis establishes an inflammatory fibroblast niche to promote NSCLC progression via the secretion of IL-6. **A** Cytokine antibody array for the supernatants of sh-NC and sh-circNOX4 CAFs, arrows indicate the cytokines with significant changes. **B** Bubble diagram of KEGG pathway analysis between sh-NC CAFs and sh-circNOX4 CAFs showed the enriched pathways. **C** The secretion level of IL-6, CCL2, TGF-β2, and IGFBP-4 in CAFs transfected with sh-NC or sh-circNOX4. **D** Schematic illustration of the coculture system of CAFs and cancer cells. **E** The secretion level of IL-6 in the indicated coculture system of CAFs and A549. Con, control.** F** The secretion level of IL-6 in the indicated coculture system of CAFs and PC9. Con, control.** G** Spearman’s correlation between FAP and IL-6 in the LUAD and LUSC cohorts from the TCGA database. **H** GSEA of affected signatures in NSCLC from the TCGA database (FAP high vs. FAP low). **I** The effect of the circNOX4/FAP axis on the secretion level of IL-6. **J** The treatment with neutralizing IL-6 antibody partly reversed the effects of circNOX4 overexpression on the CAF-mediated migration ability of NSCLC cells. **K** The treatment with neutralizing IL-6 antibody partly reversed the effects of circNOX4 overexpression on the CAF-mediated invasion ability of NSCLC cells; while the addition of rhIL-6 partly reversed the effects of circNOX4 knockdown on the CAF-mediated invasion ability of NSCLC cells. **P < 0.01, ***P < 0.001

The activation of fibroblasts coincides with paracrine signaling changes in the TME. To examine whether circNOX4 promotes metastasis in a paracrine manner, we collected supernatants from sh-NC CAFs and sh-circNOX4 CAFs and adopted a cytokine antibody array. The results showed that sh-circNOX4 CAFs secreted a decreasing panel of cytokines, including interleukin-6 (IL-6), chemokine C–C motif ligand 2 (CCL2), TGF-β2 and insulin-like growth factor binding protein 4 (IGFBP-4), which are reported as niche characteristic genes that facilitate cancer cell migration, invasion, and colonization in the metastatic process [26] (Fig. 6A and Fig. S8A). Additionally, Kyoto Encyclopedia of Genes and Genomes (KEGG) analysis showed enrichment of inflammatory signaling pathways and cytokine–cytokine receptor interaction pathways (Fig. 6B). Further validation by ELISA indicated that IL-6 was the most dramatically downregulated cytokine in sh-circNOX4 CAFs (Fig. 6C). Previous studies have revealed that IL-6, as one of the key pro-inflammatory cytokines in the TME, plays a pivotal role in initiating pro-metastatic inflammatory responses and immune cell recruitment. These results encouraged us to focus on IL-6 for further investigation.

To confirm whether IL-6 is mainly secreted by the CAFs, we initially detected the mRNA level of IL-6 in both CAFs and cancer cells. The results revealed significantly higher expression levels of IL-6 in CAFs compared to A549 and PC9 cells (Fig. S8B). Subsequently, we knocked down IL-6 in CAFs or cancer cells separately and established a transwell coculture system to measure IL-6 secretion level (Fig. 6D and Fig. S8C). ELISA analysis indicated that knockdown of IL-6 in CAFs drastically reduced its secretion within the coculture system; however, knockdown of IL-6 in A549 or PC9 only slightly lowered its secretion within the coculture system (Fig. 6E, F). We also found that IL-6 was primarily derived from the tumor stroma and fibroblast subsets in both the human and murine TMEs through bioinformatics analysis (Fig. S8D-F). Thus, these findings support that CAFs are the primary source for secreting IL-6.

Then, we determined the role of the FAP/IL-6 axis in CAF activation. Single-cell analysis from the TISCH database indicated that both FAP and IL-6 were enriched in subsets of fibroblasts (Fig. S8G). Moreover, IL-6 was positively correlated with FAP expression in the TCGA database of LUAD and LUSC cohorts (Fig. 6G). Gene set enrichment analysis (GSEA) showed substantial enrichment signatures of IL-6 signaling in NSCLC patients with high FAP expression (Fig. 6H). Importantly, the IL-6 secretion level in circNOX4-overexpressing NFs was significantly increased compared with the corresponding control, while a FAP inhibitor (linagliptin, 5 nm) hindered the CAF-secreted IL-6 level upon circNOX4 overexpression, suggesting that circNOX4 induced IL-6 secretion via FAP (Fig. 6I).

To examine whether the pro-tumoral function of circNOX4 on cancer cells was mediated by stimulating paracrine IL-6, rescue experiments involving circNOX4 and IL-6 were carried out. The wound healing assay and transwell assay together revealed that neutralizing IL-6 in a conditioned medium from circNOX4-overexpressing NFs greatly abrogated the migration and invasion capacity of NSCLC cells (Fig. 6J, K). In contrast, the exogenous addition of recombinant human IL-6 (rhIL-6) partially reversed the effects of circNOX4 knockdown on the ability of NSCLC cells to invade (Fig. 6K). Collectively, these results demonstrate that circNOX4 elicits a pro-metastatic inflammatory microenvironment by accelerating IL-6 secretion within the fibroblast niche and consequently boosts NSCLC cell migration and invasion.

### Blocking IL-6 restrains tumor progression in vivo

**Fig. 7:**
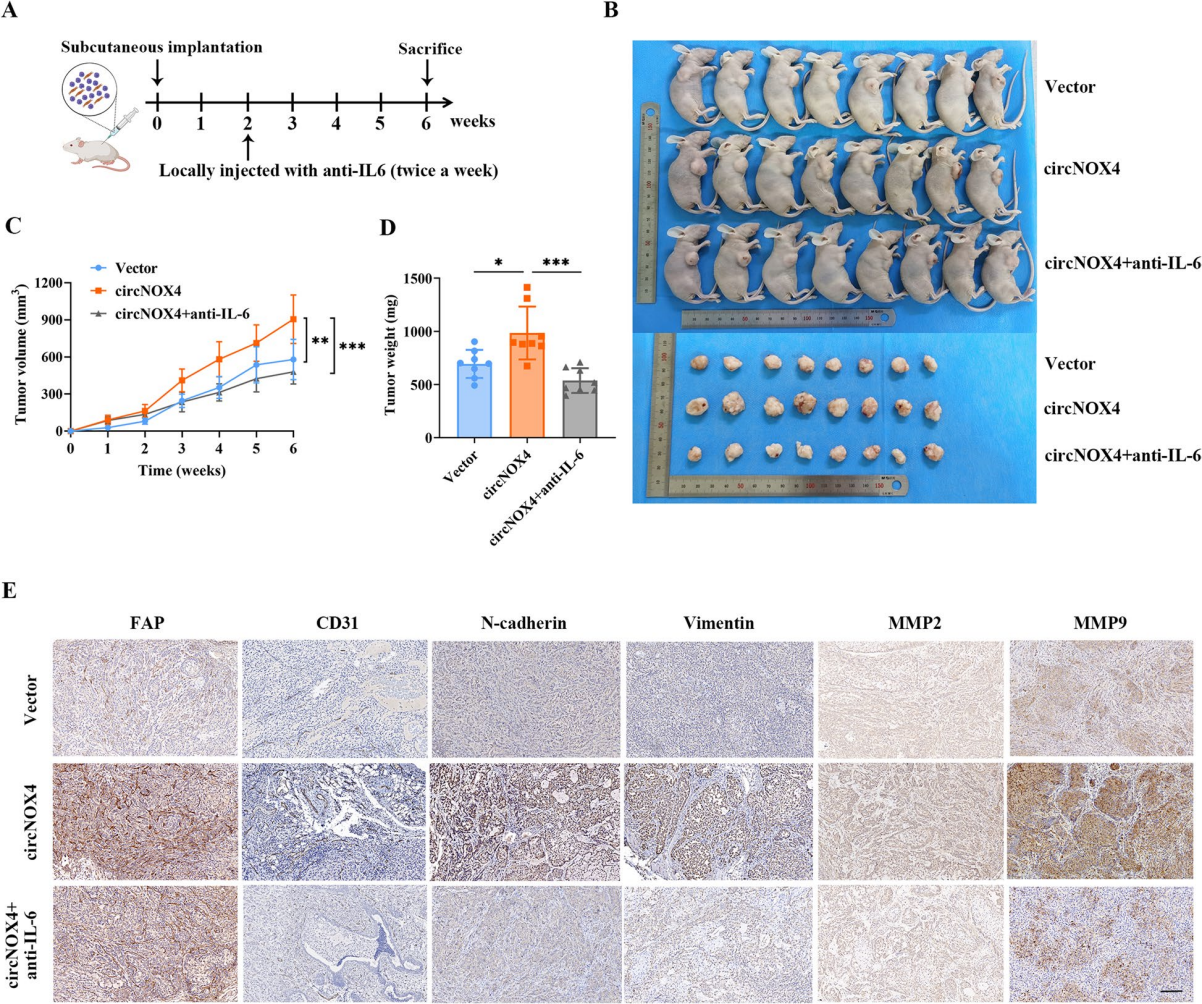
Suppression of IL-6 effectively restrains the tumor progression in vivo. **A** Schematic diagram illustrating that A549 cells were subcutaneously co-injected with NFs stably transfected with vector or circNOX4 into nude mice, and IL-6 antibody was locally injected twice a week. **B** Images of nude mice and xenograft tumors of each group (*n* = 8). **C** Tumor growth curves were shown in different treatment groups. **D** Tumor weights were measured in different treatment groups. **E** Representative images of IHC for FAP, CD31, N-cadherin, Vimentin, MMP2, and MMP9 in xenograft tumors. Scale bar = 100 μm. *P < 0.05, **P < 0.01, ***P < 0.001

**Fig. 8:**
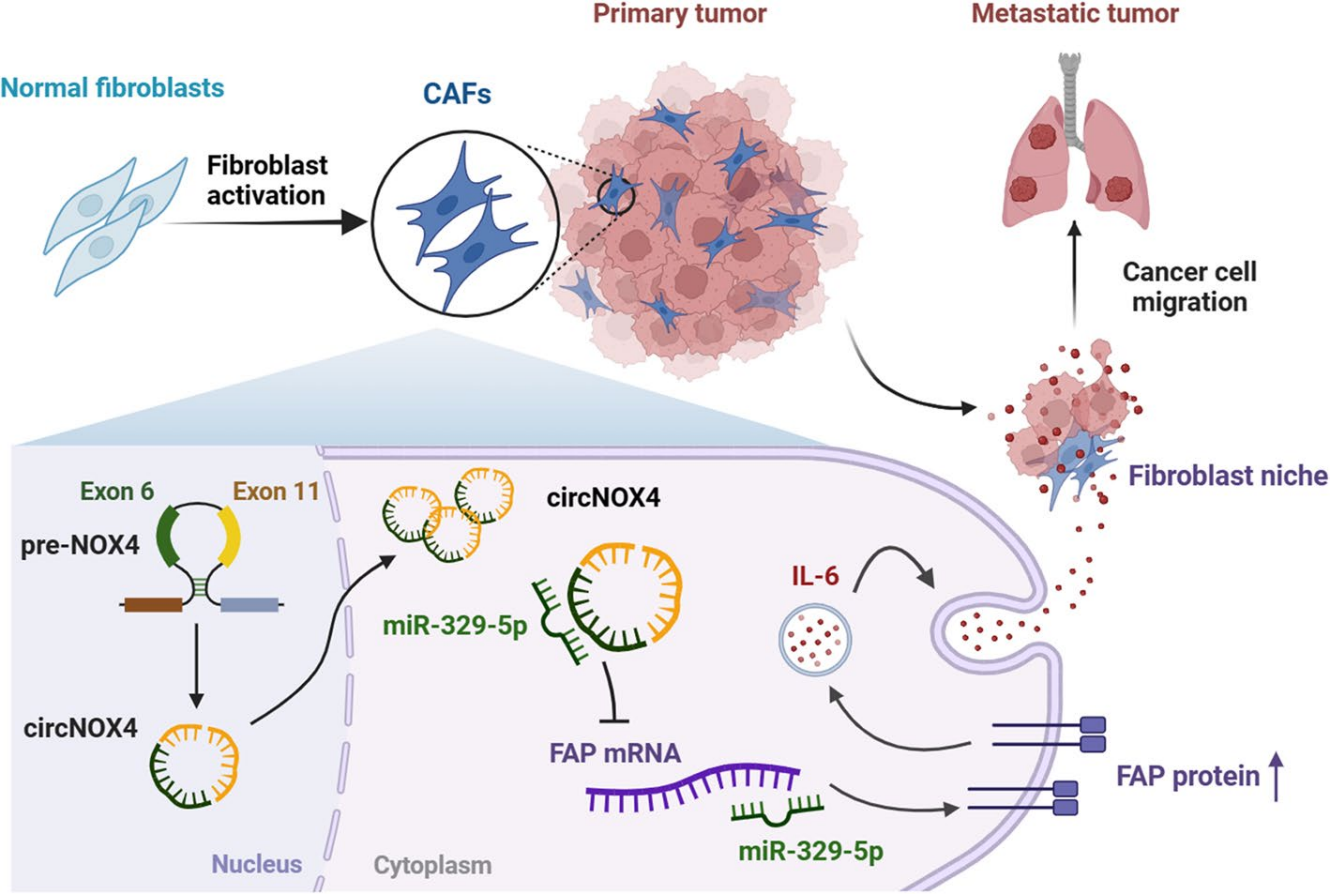
Schematic illustration visualizing the molecular mechanism of circNOX4 in activated fibroblast niche formation and NSCLC metastasis. The proposed model indicates circNOX4 is back-spliced by exons 6–11 of the NOX4 gene. The circNOX4/miR-329-5p/FAP ceRNA network in the cytoplasm reprograms the CAF phenotype and establishes an inflammatory fibroblast niche via secreting IL-6, ultimately resulting in the progression and metastasis of NSCLC

To investigate the potential therapeutic role of the circNOX4/IL-6 axis, we established xenograft tumor models and performed experimental therapy with IL-6 antibodies (Fig. 7A). A549 cells were co-injected with CAFs into the axilla of nude mice. After fourteen days after implantation, the mice received an intratumoral injection of IL-6 neutralizing antibody (2 mg/kg) twice a week. Conceivably, overexpression of circNOX4 enhanced CAF-mediated tumor growth compared to the control group. Of note, injection of the anti-IL-6 neutralizing antibody led to a reduction in tumor volume and weight (Fig. 7B-D). In further IHC analysis, the expression levels of IL-6 were increased in circNOX4-overexpressing xenograft tumors, accompanied by the increased expression of FAP, CD31, N-cadherin, Vimentin and the matrix metalloproteinases MMP2 and MMP9. However, the expression of the metastasis-related factors was partly reversed by treatment with anti-IL-6 antibody (Fig. 7E and Fig. S9A, B), indicating that IL-6 acted as a soluble mediator of remodeling the local inflammatory niche and metastatic microenvironment. In conclusion, we demonstrate that circNOX4 activates an inflammatory fibroblast niche to promote tumor progression. Our findings emphasize that the circNOX4/FAP/IL-6 axis could be exploited as a therapeutic target for CAF-based cancer therapy (Fig. 8).

## Discussion

Cancer metastasis is fostered by activated stroma, where specific microenvironments called niches are shaped [1]. Cumulative studies have highlighted the important role of CAFs in establishing these niches that facilitate tumor growth and metastasis by secreting cytokines, chemokines, growth factors, and exosomes [2]. However, how quiescent fibroblasts are educated into CAFs has not been extensively elucidated. In this study, we investigated the role and the underlying the mechanisms of FAP^+^CAF activation and inflammatory niche formation. Based on clinical samples from NSCLC patients, we found that FAP^+^CAFs correlate with distant metastasis and poor prognosis in NSCLC patients. Mechanistically, we determined that circNOX4 functions as a miR-329-5p sponge to upregulate FAP expression and induces NF to CAF phenotypic conversion. The activated CAFs subsequently enhances the production of IL-6. Since IL-6 has broad and robust effects on maintaining an inflammatory milieu and promoting cancer development, the in situ fibroblast niche boosts NSCLC metastasis and progression. Our findings highlight that targeting the circNOX4/miR-329-5p/FAP/IL-6 axis might be a novel strategy for cancer therapy in NSCLC.

In this study, we determined that FAP correlates with tumor metastasis and shortened survival of NSCLC patients. As a critical contributor to fibroblast activation, FAP is specifically expressed in the tumor stroma, which has prominent clinical implications [27, 28]. For example, FAP-specific drugs have the potential to efficaciously target lesions in cancers [29]. However, disappointing results were shown in the treatment of depleting FAP-positive cells in clinical trials of metastatic pancreatic and colorectal cancers [30, 31]. Therefore, exploring the upstream modulators of fibroblast activation is of paramount importance.

Recently, the role of circRNAs in mediating the initiation and progression of cancers has been increasingly emphasized [32]. Kristensen et al. demonstrated that circCDR1 was highly expressed in tumor stoma while its expression was absent in cancer cells, putting forward the notion that the intratumoral heterogeneity of circRNA expression should not be neglected [33]. However, the roles and biological significance of circRNAs involved in CAFs remain largely unknown. We thus explored the potential involvement of circRNAs in primary fibroblasts from human NSCLC tissues. By conducting a circRNA array, we identified a novel CAF-specific circRNA, circNOX4. Clinically, the high expression of circNOX4 in the tumor stoma was correlated with distant metastasis and worse OS in NSCLC patients. Our study links circRNA expression in CAFs with the prognosis of NSCLC patients, suggesting the therapeutic potential of targeting circNOX4 in the TME. Moreover, we found that circNOX4 was responsible for the process of fibroblast activation. Gain- and loss-of-function experiments showed that knockdown of circNOX4 “normalized” CAFs to a quiescent phenotype and downregulated the expression of FAP, while overexpression of circNOX4 in NFs was sufficient to induce an activated fibroblast phenotype with higher FAP expression. Recent studies reported that miR-30a directly targeted FAP and suppressed its expression [34]. Additionally, the transcription factor TCF21 reprogrammed FAP-high CAFs to a state that lacked pro-tumorigenic properties [13]. Our study provides novel mechanisms by which circNOX4 increases the expression of FAP, the key marker of fibroblast activation, thereby contributing to the tumor-promoting phenotypes of CAFs.

The functions of circRNAs depend on their specific subcellular locations [23]. Aiming to determine the molecular mechanisms of circNOX4, we detected its subcellular location in fibroblasts and discovered that it was mostly distributed in the cytoplasm. Cumulative studies have demonstrated that cytoplasmic circRNAs always sponge miRNAs and organize ceRNA regulatory networks to regulate gene expression [23]. By cross-referencing the prediction results of the databases, miR-329-5p was identified as one of the candidate target miRNAs of circNOX4 and was possibly involved in CAF activation. We further confirmed the circNOX4/miR-329-5p/FAP axis by conducting qRT–PCR, FISH, luciferase reporter assays, RIP, and rescue experiments. In previous studies, miR-329-5p served as either an oncogene or a tumor suppressor in different cancers [35–37]. A circRNA-mediated increase in miR-329-5p expression levels enhanced tumor proliferation and inhibited apoptosis in acute myeloid leukemia [38]. However, the expression pattern, biological function and clinical significance of miR-329-5p in the tumor stroma have not been reported. Our study provides the first evidence that the circNOX4/miR-329-5p sponge in the ceRNA network is crucial for regulating FAP expression in CAFs, indicating a promising avenue for endogenously antagonizing FAP by inhibiting circNOX4.

CAFs support cancer cells through various paracrine pathways, including the secretion of inflammatory cytokines [39]. As a versatile cytokine, IL-6 has broad and robust effects on tumor-promoting inflammation [40], the EMT process [41], and metastasis. IL-6 has also been recognized as a key marker of the inflammatory fibroblast subtype recently [42]. In this study, we identified CAFs, rather than cancer cells, are the predominant source for secreting IL-6 in the local niche. We also determined that IL-6 as the downstream mediator of the circNOX4/FAP axis in CAFs, which contributed to NSCLC metastasis. This was further confirmed by the antitumor effects seen in vivo when blocking IL-6 with a neutralizing antibody, as multiple metastatic-related factors were suppressed. Likewise, researchers found that IL-6 produced by CAFs promotes chemoresistance through the JAK-STAT3 signaling pathway and that targeting IL-6 is a strategy to improve the therapeutic efficacy of chemotherapy in gastric cancer [43]. It should be noted, however, that cytokines released by CAFs could circulate to distant organs and establish a premetastatic niche. Thus, it is possible that IL-6 fuels cancer cell dissemination from primary sites and colonization to distant organs, which requires further investigation.

## Conclusion

In summary, we unveiled a novel mechanism by which a circRNA-induced fibroblast niche contributes to the inflammatory microenvironment and metastatic progression in NSCLC. circNOX4 upregulates FAP, a crucial CAF marker, by sponging miR-329-5p. Moreover, the activated phenotype of CAFs promotes tumor growth and metastasis by enhancing IL-6 secretion and establishing an inflammatory fibroblast niche. Our findings shed new light on the therapeutic potential of targeting the circNOX4/miR-329-5p/FAP/IL-6 axis to reprogram CAFs into a quiescent state and improve clinical outcomes in NSCLC patients.

### Supplementary Information

**Additional file 1: Fig. S1.** Bioinformatics analysis of FAP in the TME. **A.** Correlation plot showing the relationship between FAP mRNA abundance and genetic changes in NSCLC from the TCGA database. **B** FAP expression with the cellular population heterogeneity in the TME as visualized by single-cell analysis from the IMMUcan database.

**Additional file 2: Fig. S2.** Isolation of primary CAFs and NFs from NSCLC patients. **A** Morphology of CAFs and NFs isolated from NSCLC samples under a light microscope. Scale bar = 200 μm. **B** Immunofluorescence staining of FAP, α-SMA, and EpCAM (epithelial marker) in CAFs. Scale bar = 50 μm. **C** Western blotting analysis of FAP and α-SMA in paired CAFs and NFs isolated from NSCLC samples (*n* = 3).

**Additional file 3: Fig. S3.** Screening and identification of the CAF-specific circRNAs.** A** Heatmap representation of differentially expressed circRNAs in human lung CAFs compared with paired NFs (*n* = 3). The up-regulated and down-regulated circRNAs are represented by the red and blue strips, respectively. **B** Construction of a competing endogenous RNA (ceRNA) regulatory network based on interactions predicted by the Targetscan, miRanda and RNAhybrid databases.** C** Identification of hsa_circ_0071486, hsa_circ_0064142, hsa_circ_0090024, hsa_circ_0023988 and hsa_circ_0053432 expression by PCR.

**Additional file 4: Fig. S4.** The effects of circNOX4 siRNA, shRNA and overexpression vector. **A** qRT-PCR analysis of circNOX4 expression in CAFs following transfection of circNOX4 siRNAs (si-circNOX4#1 and si-circNOX4#2) or negative control siRNA (si-NC), which indicated that si-circNOX4#1 yielded a better silencing effect compared with si-circNOX4#2. **B** qRT-PCR analysis of NOX4 expression in CAFs following transfection of circNOX4 siRNAs or si-NC. **C** Lentivirus vector of shRNA based on the sequence of si-circNOX4#1 was constructed. GFP-tagged lentivirus in cells were observed by fluorescence microscope. The efficiency of stably transfected circNOX4 shRNA in CAFs and overexpression vectors in NFs was detected by qRT-PCR. **D** The mRNA level of FAP in NFs transfected with vector or circNOX4. Data are presented as mean ± SD. *P < 0.05, **P < 0.01, *** *P* < 0.001.

**Additional file 5: Fig. S5.** circNOX4 is upregulated during TGF-β-induced fibroblast activation. **A** TGF-β expression in TME of NSCLC was visualized by single-cell analysis from the TISCH database, which indicated that the TGF-β signaling pathway is enriched in fibroblasts. **B** NFs were stimulated with TGF-β1 (0, 2, 5, or 10 ng/ml) for 48 h, respectively. circNOX4 and FAP expression were measured by qRT-PCR. **C** NFs were stimulated with TGF-β1 (10 ng/ml) for 0 h, 24 h, and 48 h, respectively. circNOX4 and FAP expression were measured by qRT-PCR. Data are presented as mean ± SD. *P < 0.05, **P < 0.01, *** *P* < 0.001.

**Additional file 6: Fig. S6.** Verification of the binding possibility of circNOX4 and miR-329-5p/miR-624-5p. **A** The protein-coding potential of circNOX4 predicted by circRNADb (http://reprod.njmu.edu.cn/cgi-bin/circrnadb/circRNADb.php). **B** qRT-PCR analysis of miR-624-5p expression in CAFs and matched NFs. **C** The effect of circNOX4 siRNAs on the expression of miR-624-5p in CAFs. **D** Diagram of the secondary structure of circNOX4 and the possible binding sites with miR-624-5p predicted by RNAalifold. **E** Luciferase reporter assay showing the luciferase activity of the circNOX4 luciferase reporter plasmid (WT or MUT) following transfection with miR-NC or miR-329-5p mimic into NFs. **F** Ago2-RIP assay was applied to detect the expression of circNOX4 and miR-329-5p in NFs. **G** Luciferase reporter assay showing the luciferase activity of the circNOX4 luciferase reporter plasmid (WT or MUT) following transfection with miR-NC or miR-624-5p mimic into CAFs. Data are expressed as the mean ± SD. *P < 0.05, **P < 0.01, ***P < 0.001.

**Additional file 7: Fig. S7.** circNOX4 contributes to fibroblast activation and progression of NSCLC in vivo. **A** The weight of subcutaneous xenograft tumors (*n* = 6). **B** Representative images of HE staining in xenograft tumors. Scale bar = 100 μm. **C** H-scores of FAP, CD31, N-cadherin, Vimentin, MMP2, and MMP9 by IHC analysis from mouse xenografts inoculated with indicated cells. Data are expressed as the mean ± SD. *P < 0.05, **P < 0.01, ***P < 0.001.

**Additional file 8: Fig. S8.** IL-6 expression pattern in the TME. **A** Plot showing the sums of the secretion levels of cytokines regulated by circNOX4 in CAFs. **B** IL-6 mRNA level in CAFs, A549 and PC9. **C** qRT-PCR analysis of IL-6 expression in CAFs, A549 and PC9 following transfection of IL-6 siRNAs (si-IL-6#1, si-IL-6#2 and si-IL-6#3) or negative control siRNA (si-NC). si-IL-6#1 showed a better silencing effect and was chosen for subsequent experiments. **D** The association of IL-6 and stromal score analyzed in the LUAD and LUSC cohorts from the TCGA database suggested IL-6 is closely related to the tumor stroma. **E** IL-6 expression in TME of NSCLC as visualized by single-cell analysis from the TISCH database, which indicated that IL-6 is enriched in fibroblasts. **F** Single-cell analysis of IL-6 expression in mouse lung from the Tabula Muris database. **G** The expression patterns of FAP and IL-6 in the TME of NSCLC from the TISCH database.

**Additional file 9: Fig. S9.** Treatment of the mice with anti-IL-6 neutralizing antibody stunts tumor progression in vivo. **A** Representative IHC images (left) and quantification (right) of IL-6 in xenograft tumors. Scale bar = 100 μm. **B** H-scores of FAP, CD31, N-cadherin, Vimentin, MMP2, and MMP9 by IHC analysis from mouse xenografts inoculated with indicated cells. Data are expressed as the mean ± SD. *P < 0.05, **P < 0.01, ***P < 0.001.

**Additional file 10.**

## Data Availability

Data and materials are available upon reasonable request.

## Abbreviations

CAFs: Cancer-associated fibroblasts
NFs: Normal fibroblasts
NSCLC: Non-small cell lung cancer
LUAD: Lung adenocarcinoma
LUSC: Lung squamous cell carcinoma
OS: Overall survival
TME: Tumor microenvironment
FAP: Fibroblast activation protein
circRNA: Circular RNA
miRNA: MicroRNA
IL-6: Interleukin-6
CCL2: Chemokine C–C motif ligand 2
TGF-β: Transforming growth factor-beta
IGFBP-4: Insulin-like growth factor binding protein-4
MMP2: Matrix metallopeptidase 2
MMP9: Matrix metallopeptidase 9
EMT: Epithelial-mesenchymal transition
qRT-PCR: Real-time quantitative polymerase chain reaction
FISH: Fluorescence in situ hybridization
CAF-CM: CAF-derived conditioned medium
NF-CM: NF-derived conditioned medium
CCK-8: Cell counting kit-8
IF: Immunofluorescence
ceRNAs: Competitive endogenous RNAs
siRNA: Small interfering RNA
shRNA: Short hairpin RNA
UTR: Untranslated region
FBS: Fetal bovine serum
Ago2: Argonaute-2
RISC: RNA-induced silencing complex
IHC: Immunohistochemistry
RIP: RNA binding protein immunoprecipitation
GSEA: Gene set enrichment analysis
PET: Positron emission tomography
