# Supplementary figures and images for: circNOX4 activates an inflammatory fibroblast niche to promote tumor growth and metastasis in NSCLC via FAP/IL-6 axis
Source: Mol Cancer. 2024 Mar 8;23:47. doi: 10.1186/s12943-024-01957-5 (PMC10921747; doi:10.1186/s12943-024-01957-5)

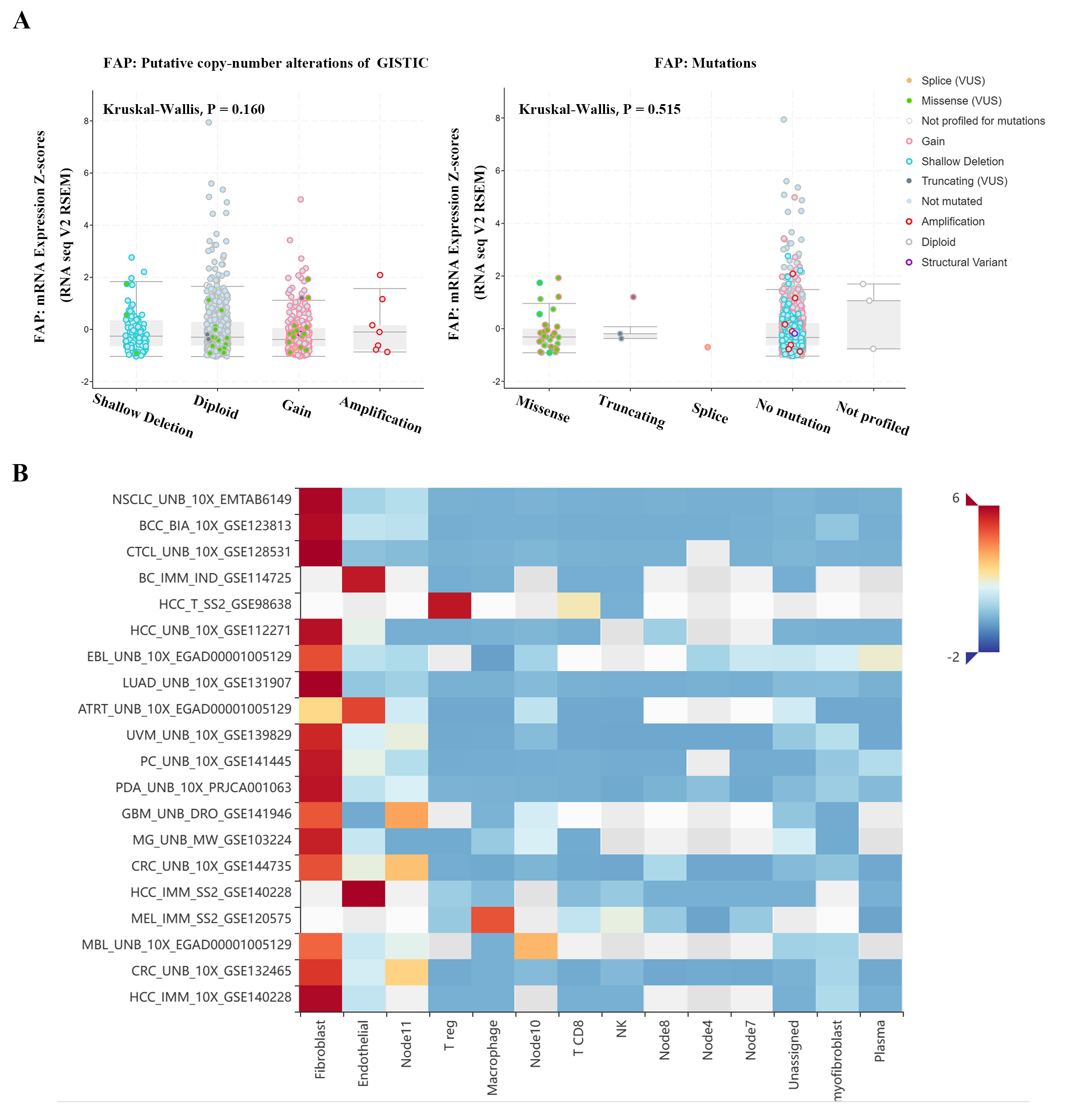

Supplement: Supplementary file 1 — Additional file 1: Fig. S1. Bioinformatics analysis of FAP in the TME. A. Correlation plot showing the relationship between FAP mRNA abundance and genetic changes in NSCLC from the TCGA database. B FAP expression with the cellular population heterogeneity in the TME as visualized by single-cell analysis from the IMMUcan database. [file 12943_2024_1957_MOESM1_ESM.tif]

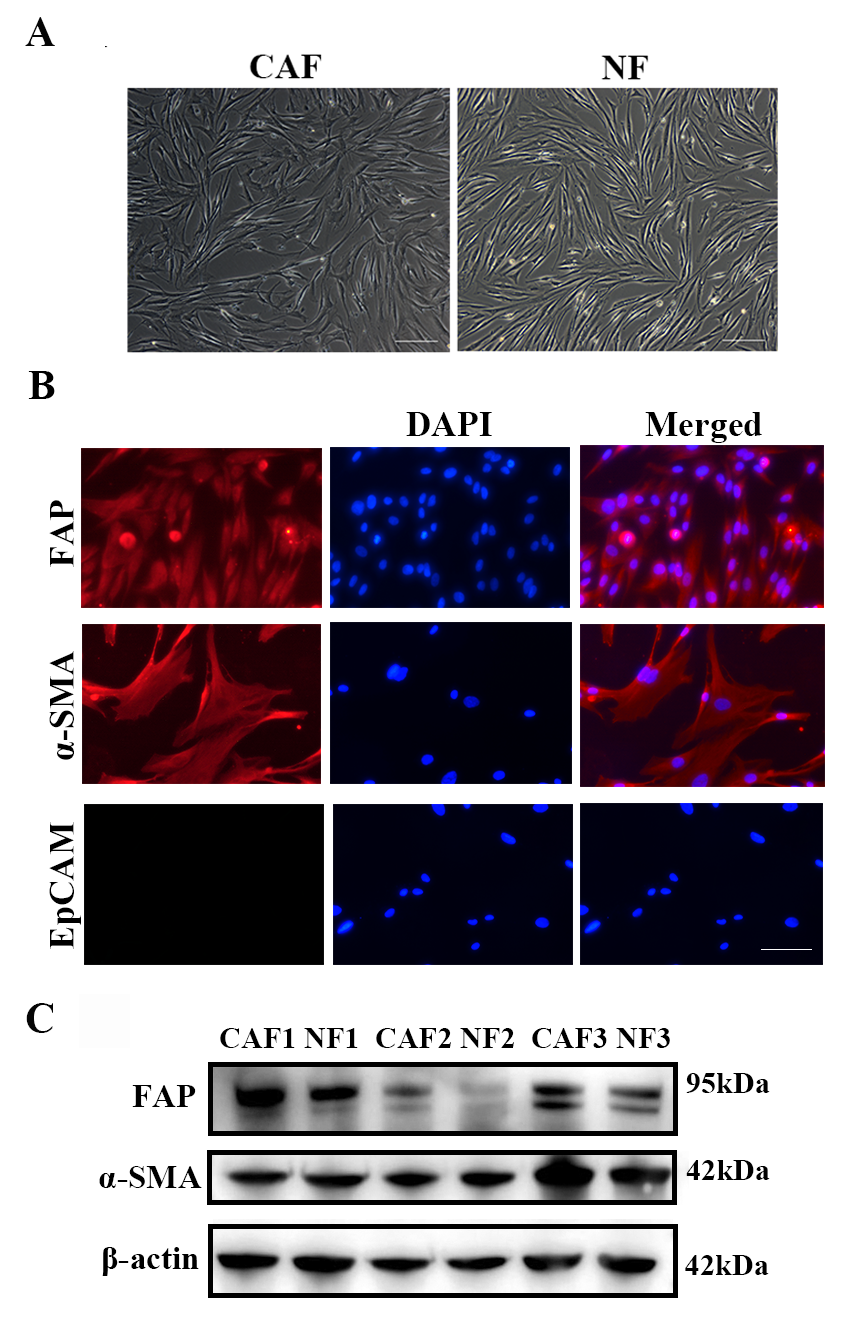

Supplement: Supplementary file 2 — Additional file 2: Fig. S2. Isolation of primary CAFs and NFs from NSCLC patients. A Morphology of CAFs and NFs isolated from NSCLC samples under a light microscope. Scale bar = 200 μm. B Immunofluorescence staining of FAP, α-SMA, and EpCAM (epithelial marker) in CAFs. Scale bar = 50 μm. C Western blotting analysis of FAP and α-SMA in paired CAFs and NFs isolated from NSCLC samples (n = 3). [file 12943_2024_1957_MOESM2_ESM.tif]

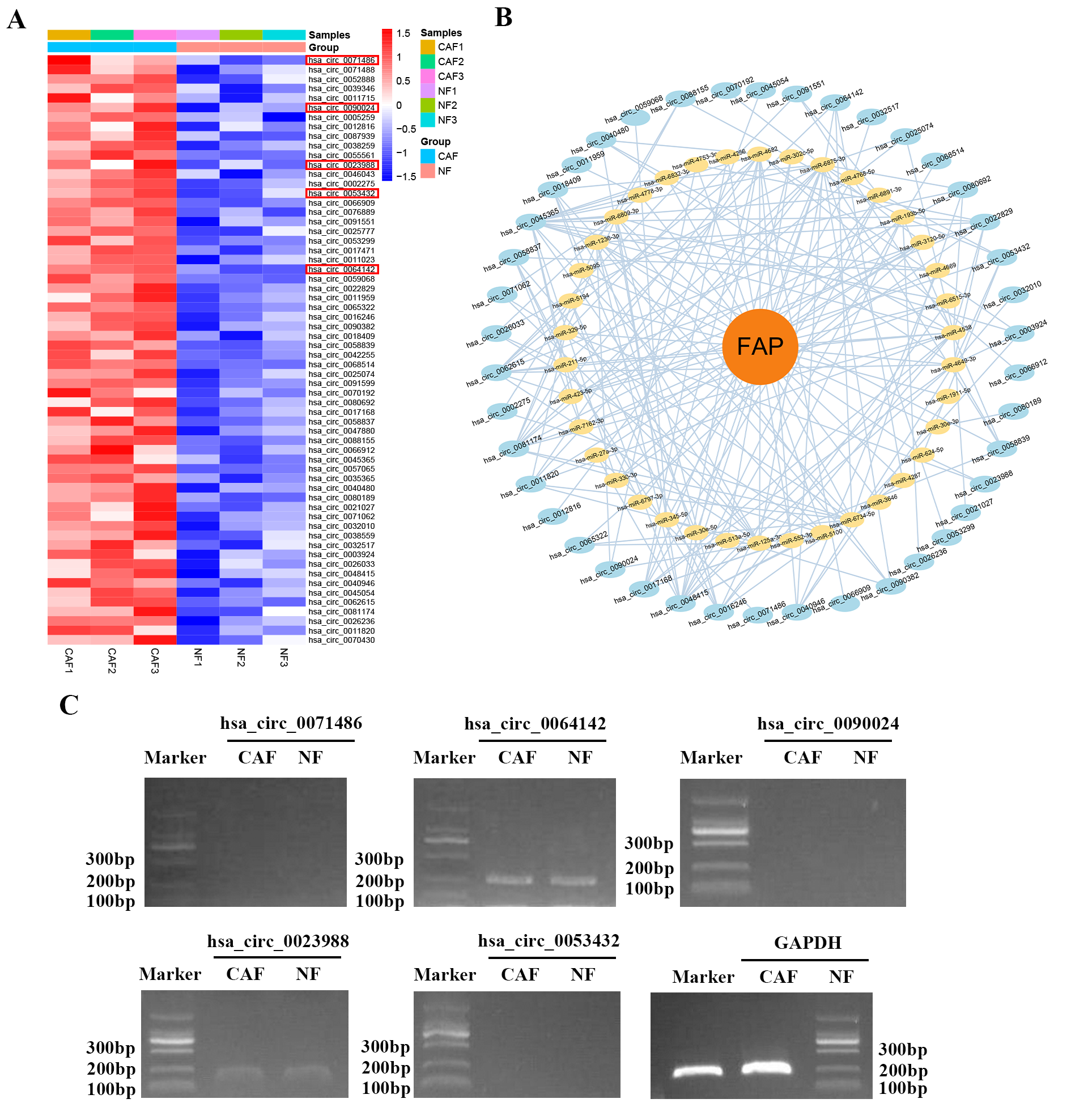

Supplement: Supplementary file 3 — Additional file 3: Fig. S3. Screening and identification of the CAF-specific circRNAs. A Heatmap representation of differentially expressed circRNAs in human lung CAFs compared with paired NFs (n = 3). The up-regulated and down-regulated circRNAs are represented by the red and blue strips, respectively. B Construction of a competing endogenous RNA (ceRNA) regulatory network based on interactions predicted by the Targetscan, miRanda and RNAhybrid databases. C Identification of hsa_circ_0071486, hsa_circ_0064142, hsa_circ_0090024, hsa_circ_0023988 and hsa_circ_0053432 expression by PCR. [file 12943_2024_1957_MOESM3_ESM.tif]

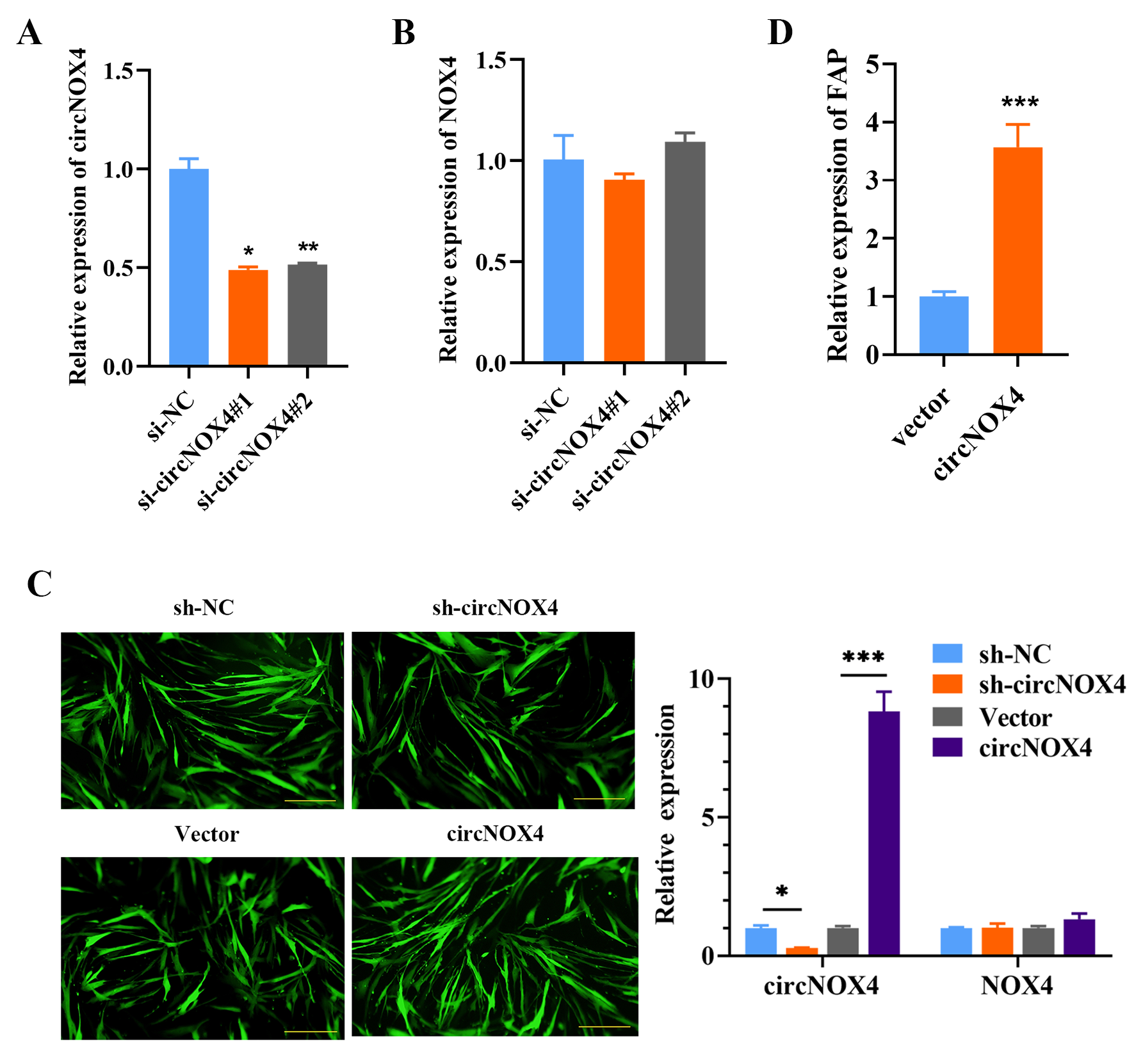

Supplement: Supplementary file 4 — Additional file 4: Fig. S4. The effects of circNOX4 siRNA, shRNA and overexpression vector. A qRT-PCR analysis of circNOX4 expression in CAFs following transfection of circNOX4 siRNAs (si-circNOX4#1 and si-circNOX4#2) or negative control siRNA (si-NC), which indicated that si-circNOX4#1 yielded a better silencing effect compared with si-circNOX4#2. B qRT-PCR analysis of NOX4 expression in CAFs following transfection of circNOX4 siRNAs or si-NC. C Lentivirus vector of shRNA based on the sequence of si-circNOX4#1 was constructed. GFP-tagged lentivirus in cells were observed by fluorescence microscope. The efficiency of stably transfected circNOX4 shRNA in CAFs and overexpression vectors in NFs was detected by qRT-PCR. D The mRNA level of FAP in NFs transfected with vector or circNOX4. Data are presented as mean ± SD. *P < 0.05, **P < 0.01, *** P < 0.001. [file 12943_2024_1957_MOESM4_ESM.tif]

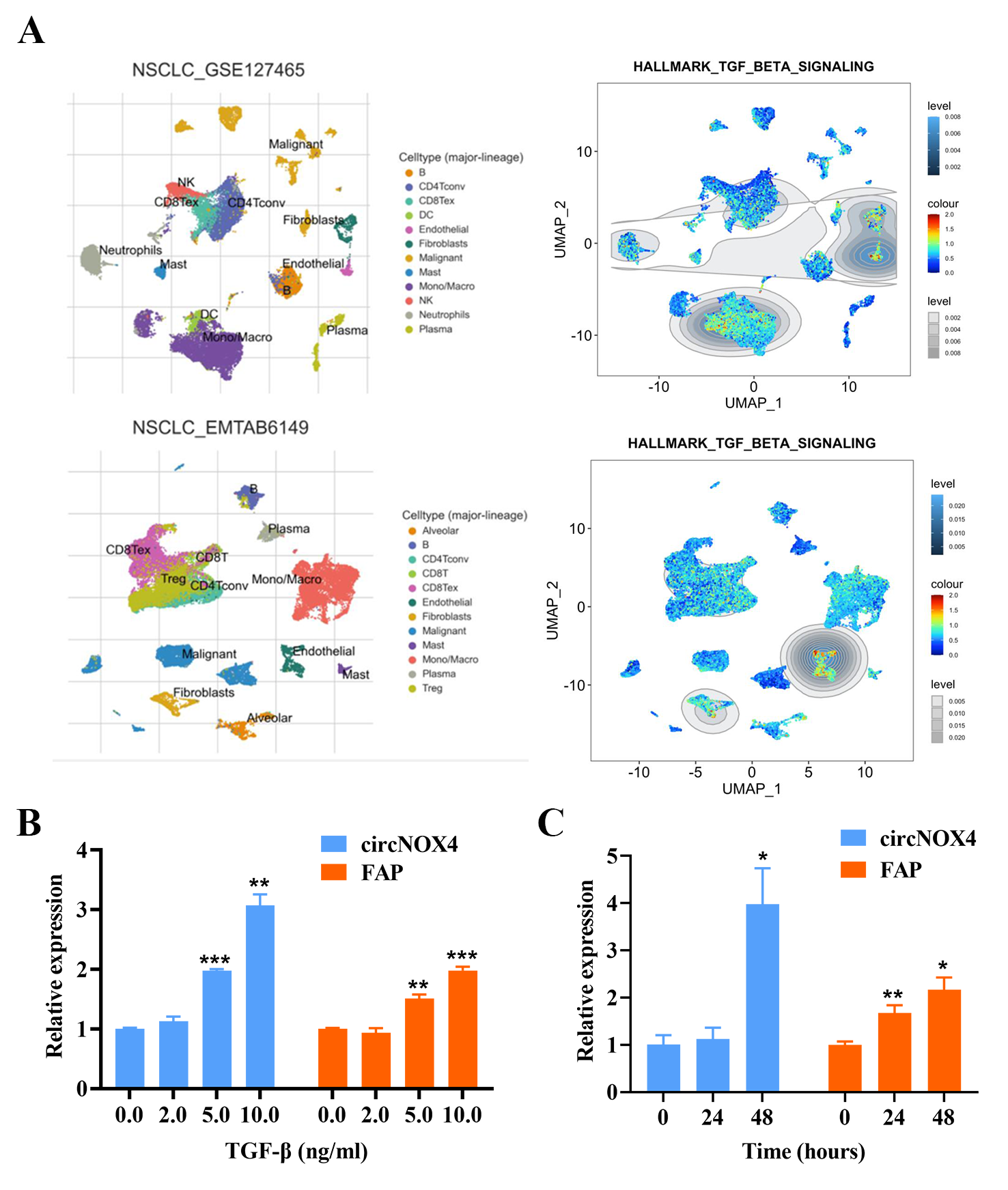

Supplement: Supplementary file 5 — Additional file 5: Fig. S5. circNOX4 is upregulated during TGF-β-induced fibroblast activation. A TGF-β expression in TME of NSCLC was visualized by single-cell analysis from the TISCH database, which indicated that the TGF-β signaling pathway is enriched in fibroblasts. B NFs were stimulated with TGF-β1 (0, 2, 5, or 10 ng/ml) for 48 h, respectively. circNOX4 and FAP expression were measured by qRT-PCR. C NFs were stimulated with TGF-β1 (10 ng/ml) for 0 h, 24 h, and 48 h, respectively. circNOX4 and FAP expression were measured by qRT-PCR. Data are presented as mean ± SD. *P < 0.05, **P < 0.01, *** P < 0.001. [file 12943_2024_1957_MOESM5_ESM.tif]

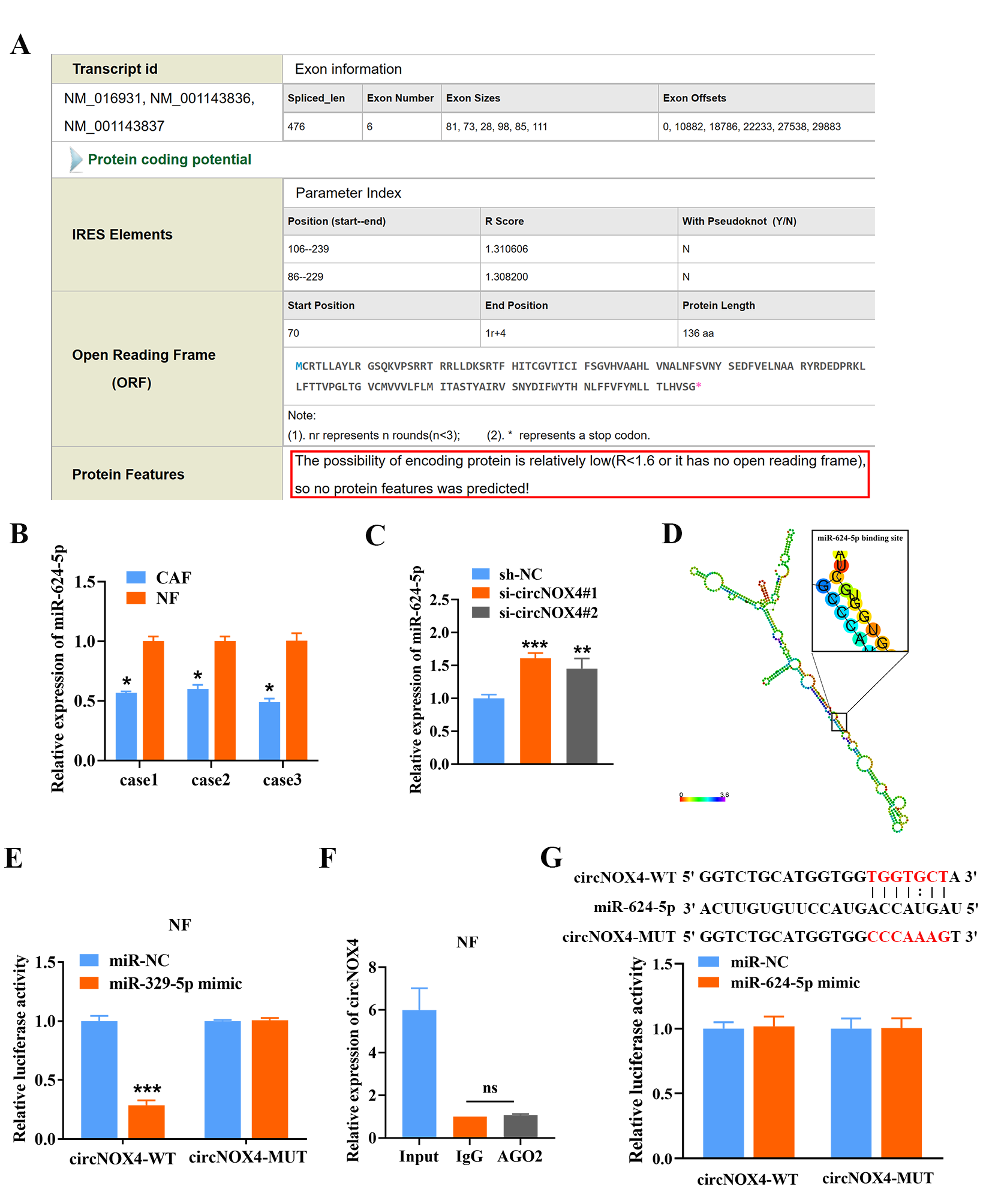

Supplement: Supplementary file 6 — Additional file 6: Fig. S6. Verification of the binding possibility of circNOX4 and miR-329-5p/miR-624-5p. A The protein-coding potential of circNOX4 predicted by circRNADb (http://reprod.njmu.edu.cn/cgi-bin/circrnadb/circRNADb.php). B qRT-PCR analysis of miR-624-5p expression in CAFs and matched NFs. C The effect of circNOX4 siRNAs on the expression of miR-624-5p in CAFs. D Diagram of the secondary structure of circNOX4 and the possible binding sites with miR-624-5p predicted by RNAalifold. E Luciferase reporter assay showing the luciferase activity of the circNOX4 luciferase reporter plasmid (WT or MUT) following transfection with miR-NC or miR-329-5p mimic into NFs. F Ago2-RIP assay was applied to detect the expression of circNOX4 and miR-329-5p in NFs. G Luciferase reporter assay showing the luciferase activity of the circNOX4 luciferase reporter plasmid (WT or MUT) following transfection with miR-NC or miR-624-5p mimic into CAFs. Data are expressed as the mean ± SD. *P < 0.05, **P < 0.01, ***P < 0.001. [file 12943_2024_1957_MOESM6_ESM.tif]

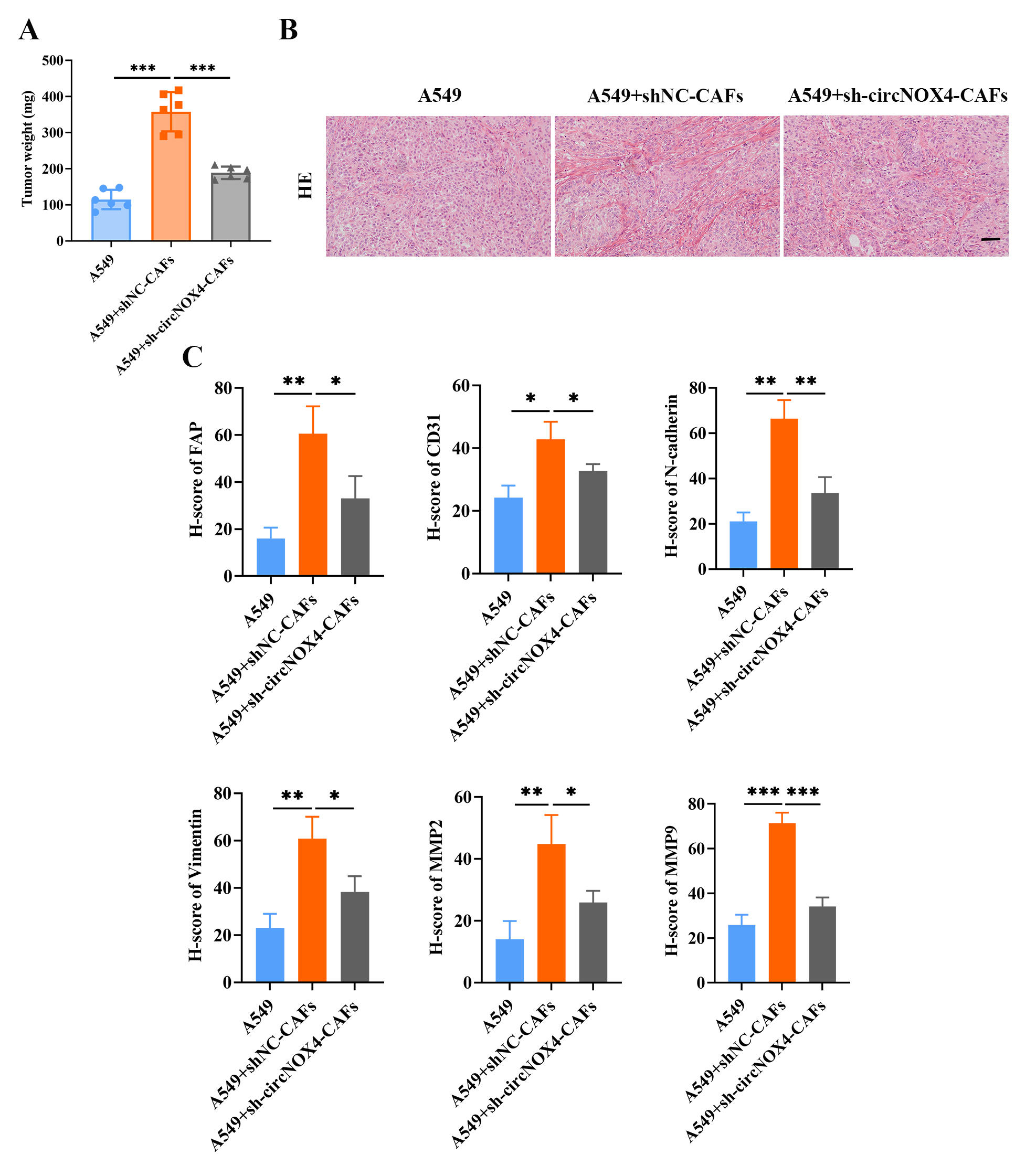

Supplement: Supplementary file 7 — Additional file 7: Fig. S7. circNOX4 contributes to fibroblast activation and progression of NSCLC in vivo. A The weight of subcutaneous xenograft tumors (n = 6). B Representative images of HE staining in xenograft tumors. Scale bar = 100 μm. C H-scores of FAP, CD31, N-cadherin, Vimentin, MMP2, and MMP9 by IHC analysis from mouse xenografts inoculated with indicated cells. Data are expressed as the mean ± SD. *P < 0.05, **P < 0.01, ***P < 0.001. [file 12943_2024_1957_MOESM7_ESM.tif]

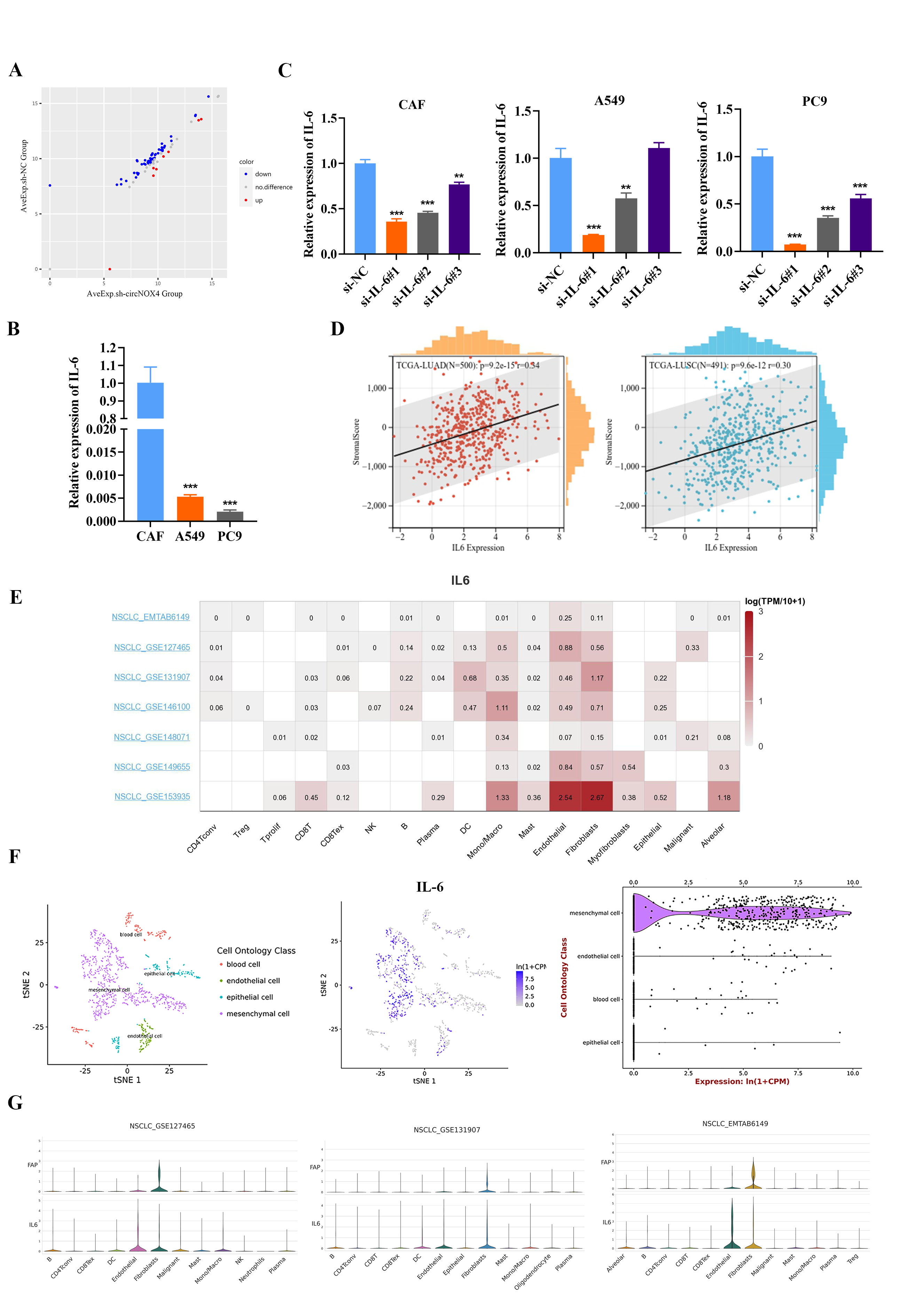

Supplement: Supplementary file 8 — Additional file 8: Fig. S8. IL-6 expression pattern in the TME. A Plot showing the sums of the secretion levels of cytokines regulated by circNOX4 in CAFs. B IL-6 mRNA level in CAFs, A549 and PC9. C qRT-PCR analysis of IL-6 expression in CAFs, A549 and PC9 following transfection of IL-6 siRNAs (si-IL-6#1, si-IL-6#2 and si-IL-6#3) or negative control siRNA (si-NC). si-IL-6#1 showed a better silencing effect and was chosen for subsequent experiments. D The association of IL-6 and stromal score analyzed in the LUAD and LUSC cohorts from the TCGA database suggested IL-6 is closely related to the tumor stroma. E IL-6 expression in TME of NSCLC as visualized by single-cell analysis from the TISCH database, which indicated that IL-6 is enriched in fibroblasts. F Single-cell analysis of IL-6 expression in mouse lung from the Tabula Muris database. G The expression patterns of FAP and IL-6 in the TME of NSCLC from the TISCH database. [file 12943_2024_1957_MOESM8_ESM.tif]

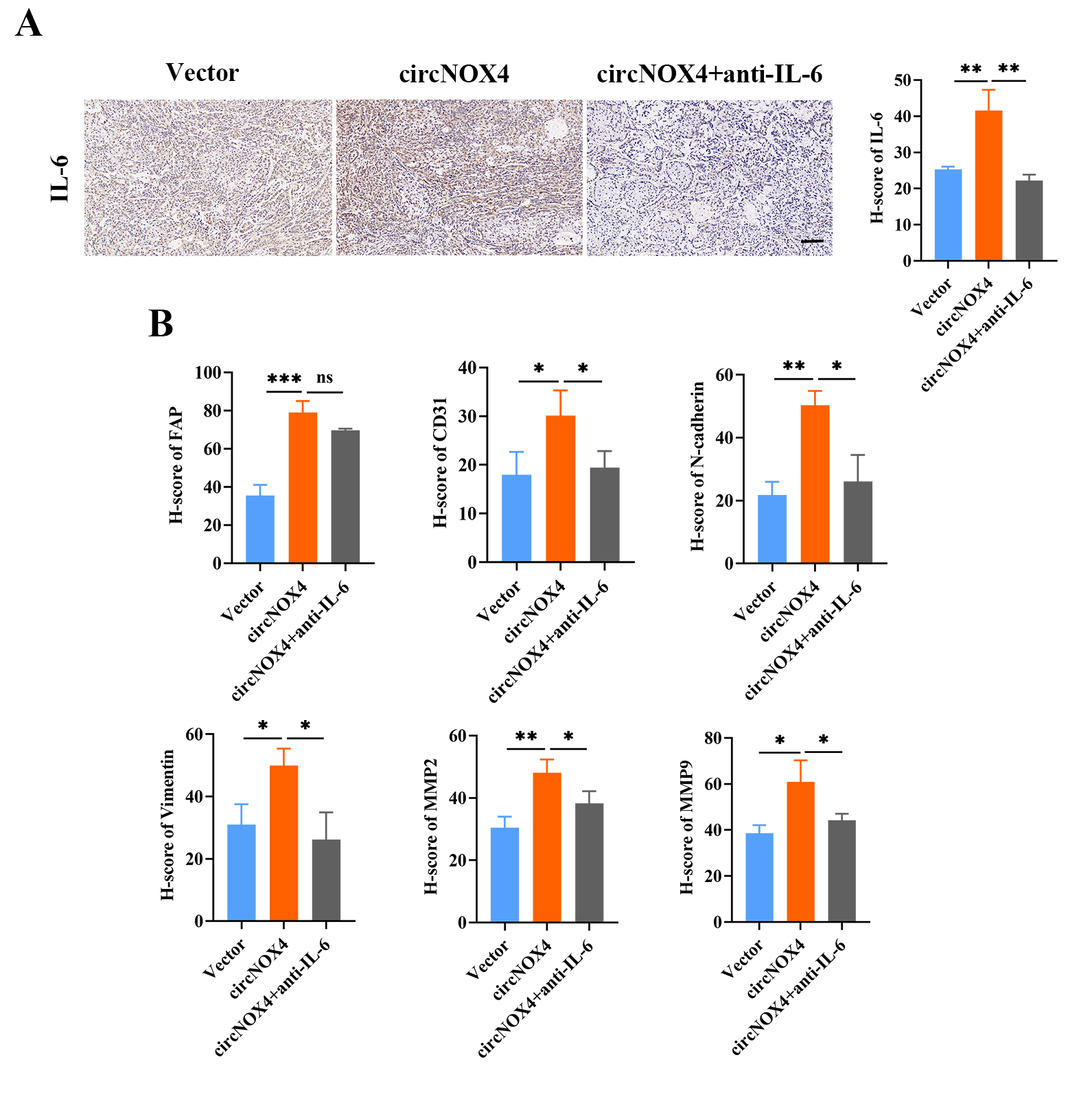

Supplement: Supplementary file 9 — Additional file 9: Fig. S9. Treatment of the mice with anti-IL-6 neutralizing antibody stunts tumor progression in vivo. A Representative IHC images (left) and quantification (right) of IL-6 in xenograft tumors. Scale bar = 100 μm. B H-scores of FAP, CD31, N-cadherin, Vimentin, MMP2, and MMP9 by IHC analysis from mouse xenografts inoculated with indicated cells. Data are expressed as the mean ± SD. *P < 0.05, **P < 0.01, ***P < 0.001. [file 12943_2024_1957_MOESM9_ESM.tif]
