# Supplementary material for: circNOX4 activates an inflammatory fibroblast niche to promote tumor growth and metastasis in NSCLC via FAP/IL-6 axis
Source: Mol Cancer. 2024 Mar 8;23:47. doi: 10.1186/s12943-024-01957-5 (PMC10921747; doi:10.1186/s12943-024-01957-5)
Supplement: Supplementary file 10 — Additional file 10. [file 12943_2024_1957_MOESM10_ESM.docx]

**Supplementary information**

**Supplementary Table 1 Information of NSCLC patients for fibroblast isolation/circRNA array**

| **Sample**  **ID** | **Age** | **Sex** | **Clinical stage** | **T stage** | **N stage** | **M stage** | **Histological type** |
| --- | --- | --- | --- | --- | --- | --- | --- |
| 1 | 52 | Female | IA2 | T1b | N0 | M0 | LUAD |
| 2 | 54 | Male | IIIA | T1b | N2 | M0 | LUAD |
| 3 | 67 | Female | IB | T2a | N0 | M0 | LUAD |
| 4 | 69 | Male | IB | T2a | N0 | N0 | LUAD |
| 5 | 53 | Female | IIIA | T4 | N0 | M0 | LUAD |

**Supplementary Table 2. List of antibodies used this study**

| **Antigen** | **Vendor** | **Cat.No** | **Application** | **dilution** |
| --- | --- | --- | --- | --- |
| FAP | Abcam | ab207178 | IHC; WB | 1:250; 1:1000 |
| FAP | Bioss | Bs-5758R | IF | 1:100 |
| α-SMA | Abcam | ab124964 | IF;WB | 1:100; 1:1000 |
| EpCAM | Abcam | ab71916 | IF | 1:100 |
| CoraLite488- conjugated Affinipure Goat Anti-Rabbit IgG(H+L) | Proteintech | SA00013-2 | IF | 1:500 |
| Goat Anti-Rabbit IgG(H+L), CoraLite594 conjugate | Proteintech | SA00013-4 | IF | 1:500 |
| AGO2 | Abcam | ab186733 | RIP | 1:50 |
| lgG | Abcam | ab172730 | RIP | 1:100 |
| MMP9 | Abcam | ab76003 | IHC | 1:200 |
| MMP2 | Abcam | ab86607 | IHC | 1:200 |
| IL-6 | Servicebio | GB11117 | IHC | 1:200 |
| CK19 | Servicebio | CB11197 | IF | 1:200 |
| N-cadherin | Servicebio | GB111273 | IHC | 1:100 |
| CD31 | Proteintech | 28083-1-AP | IHC | 1:200 |
| Vimentin | Proteintech | 10366-1-AP | IHC | 1:400 |
| Goat Anti-mouse IgG (HRP) | Servicebio | GB23301 | IHC | 1:200 |

**Supplementary Table 3. Sequences of primers in this study**

| circNOX4 (hsa_circ_0023988) | Divergent Forward | CGTTGCATGTTTCAGGCTAGG | 155bp |
| --- | --- | --- | --- |
|  | Divergent Reverse | ACAATCTCCTGGTTCTCCTGCT |  |
|  | Convergent Forward | TACCCATGTGCCGAACACTC | 143bp |
|  | Convergent Reverse | CACATGCACGCCTGAGAAAA |  |
| hsa_circ_0064142 | Forward | AGTAGAACGACTCCGAGAAGGA | 195bp |
|  | Reverse | GGTGCTGAGTTCTAGTCTCTTGG |  |
| hsa_circ_0071486 | Forward | AGGCCAAGTACTGACTGCTG | 299bp |
|  | Reverse | AGGACAACCCTCGATAGTGC |  |
| hsa_circ_0090024 | Forward | CCCGGAGAGAGATAGCAGAAAC | 113bp |
|  | Reverse | TCATGGACATAAAGAAAACAACACC |  |
| hsa_circ_0053432 | Forward | TCAAAGCAGGATGATGTGGTTC | 199bp |
|  | Reverse | GGGGGAGAAGCAGGGACTTA |  |
| NOX4 | Forward | AAACACCTCTGCCTGTTCATCT | 286bp |
|  | Reverse | GGTTTCAGTTGGACACCCCAA |  |
| FAP | Forward | GAATGTTTCGGTCCTGTCTATATGT | 196bp |
|  | Reverse | GTGAATATGTTTGTAGCCATCCTTG |  |
| GAPDH | Divergent Forward | CCCTGTGCTCAACCAGCTCTC | 111bp |
|  | Divergent Reverse | CCGACCTTCACCTTCCCCAT |  |
|  | Convergent Forward | GGAGTCCACTGGCGTCTTCA | 240bp |
|  | Convergent Reverse | GTCATGAGTCCTTCCACGATACC |  |
| α-SMA | Forward | GGTGGGAATGGGACAAAAAGAC | 284bp |
|  | Reverse | CCGCCTGGATAGCCACATAC |  |
| U6 | Forward | CTCGCTTCGGCAGCACA | 94bp |
|  | Reverse | AACGCTTCACGAATTTGCGT |  |
| hsa-miR-329-5p | Forward | ACACTCCAGCTGGGGAGGTTTTCTGGGTTTC | 67bp |
|  | Reverse | TGGTGTCGTGGAGTCG |  |
| hsa-miR-624-5p | Forward | ACACTCCAGCTGGGTAGTACCAGTACCTTG | 66bp |
|  | Reverse | TGGTGTCGTGGAGTCG |  |
| MMP2 | Forward | TGTTGGTGGGAACTCAGAAGGT | 281bp |
|  | Reverse | GACGGAAGTTCTTGGTGTAGGTGT |  |
| MMP14 | Forward | CCTGCGTCCATCAACACTGC | 230bp |
|  | Reverse | TCTTCGTTGAAACGGTAGTACTTG |  |
| CCL2 | Forward | GATCTCAGTGCAGAGGCTCG | 155bp |
|  | Reverse | TTTGCTTGTCCAGGTGGTCC |  |
| COL1A1 | Forward | CCCCTGGAAAGAATGGAGATG | 104bp |
|  | Reverse | AGCTGTTCCGGGCAATCCT |  |
| PDGFRβ | Forward | TGACTGACTTCCTCTTGGATATGC | 200bp |
|  | Reverse | AAATTGTAGTGTGCCCACCTCTC |  |
| IL-6 | Forward | GTCCAGTTGCCTTCTCCCTG | 168bp |
|  | Reverse | CTGAGATGCCGTCGAGGATG |  |

**Supplementary Table 4. Probes for FISH**

| circNOX4 | Sense (5’-3’) | CTTAGACACAATCCTAGCCTGAAACATGCAACG |
| --- | --- | --- |
| miR-329-3p | Sense (5’-3’) | GAAACAGAAACCCAGAAAACCTC |

**Supplementary Table 5. Sequences of RNA nucleotides in this study**

| si-circNOX4#1 | Sense (5’-3’) | GUUUCAGGCUAGGAUUGUGdTdT |
| --- | --- | --- |
| si-circNOX4#2 | Sense (5’-3’) | GCAUGUUUCAGGCUAGGAUdTdT |
| siRNA negative control | Sense (5’-3’) | UUCUCCGAACGUGUCACGUdTdT |
| sh-NC | Sense (5’-3’) | GTTCTCCGAACGTGTCACGT |
| sh-circNOX4 | Sense (5’-3’) | GTTTCAGGCTAGGATTGTG |
| hsa-miR-329-5p mimic | Sense (5’-3’) | GAGGUUUUCUGGGUUUCUGUUUC |
| hsa-miR-329-5p inhibitor | Sense (5’-3’) | GAAACAGAAACCCAGAAAACCUC |
| hsa-miR-624-5p mimic | Sense (5’-3’) | UAGUACCAGUACCUUGUGUUCA |
| hsa-miR-624-5p inhibitor | Sense (5’-3’) | UGAACACAAGGUACUGGUACUA |
| mimic NC | Sense (5’-3’) | CAGUACUUUUGUGUAGUACAAA |
| inhibitor NC | Sense (5’-3’) | UUUGUACUACACAAAAGUACUG |
| si-IL6#1 | Sense (5’-3’) | GGAGUUUGAGGUAUACCUAdTdT |
| si-IL6#1 | Sense (5’-3’) | GCUGUGCAGAUGAGUACAAdTdT |
| si-IL6#1 | Sense (5’-3’) | GCAGCUUUAAGGAGUUCCUdTdT |

**Supplementary Table 6. The sequences of wildtype and mutant form of circNOX4 in the luciferase reporter assay**

Wildtype form of circNOX4:

ctaggattgtgtctaagc**AGAGCCTC**agcatctgttcttaacctcaactgcagccttatccttttacccgctgcccatctggtgaatgccctcaacttctcagtgaattacagt**GAAGACTTT**gttgaactgaatgcagcaagataccgagatgaggat**CCTAGAAAACTTC**tcttcacaactgttcctggcctgacaggggtctgc

Bold: the wildtype target site of miR-329-5p

Wildtype form of circNOX4:

gcaagataccgagatgaggatcctagaaaacttctcttcacaactgttcctggcctgacaggggtctgcatggtgg**TGGTGCTA**ttcctcatgatcacagcctctacatatgcaataagagtttctaactatgatatc

Bold: the wildtype target site of miR-624-5p

Mutant form of circNOX4:

ctaggattgtgtctaagc**AGAGCCTC**agcatctgttcttaacctcaactgcagccttatccttttacccgctgcccatctggtgaatgccctcaacttctcagtgaattacagt**GAAGACTTT**gttgaactgaatgcagcaagataccgagatgaggat**CCTAGAAAACTTC**tcttcacaactgttcctggcctgacaggggtctgc

Bold: the mutant target site of miR-329-5p

Mutant form of circNOX4:

gcaagataccgagatgaggatcctagaaaacttctcttcacaactgttcctggcctgacaggggtctgcatggtgg**CCCAAAGT**ttcctcatgatcacagcctctacatatgcaataagagtttctaactatgatatc

Bold: the mutant target site of miR-624-5p

**Supplementary Table 7.The sequence of wildtype and mutant form of FAP 3’UTR in the luciferase reporter assay**

Wildtype form of FAP 3’UTR:

aaaacgatgcagatgc**AAGCCT**gtatc**AGAATCTGAAAACCTT**atataaacccctcagacagtttgcttattttattttttatgttgtaaaatgctagtataaacaaacaaattaatgttgttctaaaggctgttaaa

Bold: the wildtype target site of miR-329-5p

Mutant form of FAP 3’UTR:

aaaacgatgcagatgc**TTCGGA**gtatc**TCTTAAGATTGTTGGC**atataaacccctcagacagtttgcttattttattttttatgttgtaaaatgctagtataaacaaacaaattaatgttgttctaaaggctgttaaa

Bold: the mutant target site of miR-329-5p
